# The expression and role of SUZ12 in lung adenocarcinoma

**DOI:** 10.1002/cam4.70190

**Published:** 2024-10-13

**Authors:** Xingsheng Hu, Chunhong Hu, Ping Zhong

**Affiliations:** ^1^ Department of Oncology Affiliated Hospital of North Sichuan Medical College Nanchong China; ^2^ Department of Oncology the Second Xiangya Hospital of Central South University Changsha China; ^3^ Department of Dermatology Beijing Anzhen Hospital Affiliated to Capital Medical University, Nanchong Hospital (Nanchong Central Hospital and The Second Clinical Medical School of North Sichuan Medical College) Nanchong China

**Keywords:** epigenetics, lung adenocarcinoma, polycomb repressive complex 2, SUZ12, transcription factor

## Abstract

**Background:**

SUZ12 is one of the core members of the polycomb repressive complex 2 (PRC2), but its expression and role in lung adenocarcinoma (LUAD) are unclear. We aimed to explore the expression, prognosis, biological functions and roles of SUZ12 in LUAD.

**Methods:**

The expression of SUZ12 was detected by immunohistochemical staining, qRT‐PCR, and western blotting in LUAD tissues and cells. The biological functions and molecular mechanisms of SUZ12 were characterized by a range of in vitro and in vivo experiments.

**Results:**

SUZ12 was overexpressed in LUAD tissues, and high SUZ12 expression was correlated with worse clinicopathological features and a poorer prognosis. Knockdown of SUZ12 significantly inhibited cell growth, colony formation, invasion, and migration, and induced apoptosis and G1/S phase arrest, while overexpression of SUZ12 had the opposite effects. Knockdown of SUZ12 decreased the tumorigenic capacity of A549 cells in vivo. The expression of key signaling molecules related to the cell cycle, apoptosis, migration, and immunity were altered by the knockdown or overexpression of SUZ12. SUZ12 can directly bind to the Bax promoter region, EZH2 and H3K27me3 levels dependents on SUZ12. The expression levels of SUZ12 and Bax were negatively correlated in LUAD tissues.

**Conclusions:**

SUZ12 is a new oncogene related to the poor prognosis of LUAD. SUZ12 regulates LUAD progression by regulating the expression of related signaling molecules, and as a part of the PRC2 complex, it may bind to the Bax promoter to silence Bax expression.

## 1 INTRODUCTION

According to global cancer statistics, lung cancer ranked second (11.4% of new cases) in cancer incidence and first (18.0% of deaths) in mortality in 2020.^1^

Lung adenocarcinoma (LUAD) is the most common type of non‐small cell lung cancer (NSCLC), accounting for approximately 50% of cases,^2^ and its overall 5‐year survival rate is only about 15%.^2^ The pathogenesis of LUAD is complex, and it presents high heterogeneity; molecular changes are the main cause of the disease. Although mutations in driver genes have been identified, these changes only account for a fraction of all pathogenic factors. Therefore, continued exploration of the underlying molecular mechanisms is the main research focus.

Epigenetic modifications strongly affect the transmission of genetic information, and its role in the pathogenesis of cancer has been increasingly recognized and affirmed.^3^ Epigenetic modifications change gene expression at the transcriptional and translational levels by changing the structure of DNA, chromatin and RNA stability without causing DNA sequence changes.^4^ The polycomb group is one of the key regulatory families in epigenetics, and its main mechanism involves silencing gene expression via posttranscriptional modification.^3^ This family mainly consists of two core protein complexes, polycomb repressive complex 1 (PRC1) and polycomb repressive complex 2 (PRC2).^5^ SUZ12 is one of the three core members of PRC2 (EZH2, SUZ12, and EED). Its main function is to maintain the stability of PRC2, which is indispensable for the latter.^5^ SUZ12 expression was reported to be up‐regulated in head and neck squamous carcinoma (HNSCC), ovarian cancer, gastric cancer tissues etc., and correlated with a poor prognosis.^6^, ^7^, ^8^ In NSCLC, SUZ12 mRNA was overexpressed in cancer tissues and associated with tumor size, lymphatic metastasis etc.,^9^ but its prognostic in LUAD is unclear. SUZ12 was also reported to play an important role in facilitating proliferation,^6^, ^8^ migration,^6^, ^8^ G1/S phase transition,^10^ but arresting apoptosis.^7^, ^8^ In terms of role of molecule mechanisms, some studies reported that SUZ12 may play a pro‐cancer role by downregulating the expression of p21^11^ and p27.^12^ However, other underlying mechanisms of SUZ12 are unclear. The aims of this study was to explore the expression, prognosis, biological functions, and roles of SUZ12 in LUAD.

## 2 MATERIALS AND METHODS

### 2.1 Bioinformatical analyses

UALCAN (https://ualcan.path.uab.edu/cgi‐bin/ualcan‐res.pl) online tool was used to analyze SUZ12 expression in LUAD tissue and normal lung tissue from The Cancer Genome Atlas (TCGA) database. Kaplan–Meier Plotter (https://kmplot.com/analysis/index.php?p=background) online tool was used to analyzed SUZ12 prognosis in LUAD from TCGA database.

### 2.2 Patients and tissue specimens

Paraffin‐embedded tissues were collected from LUAD patients who underwent surgical resection at the Second Xiangya Hospital of Central South University between January 2018 and May 2019. All patients had definite pathological diagnoses and follow‐up data. Patient data, including sex, age, smoking status, histological type, differentiation degree, and pTNM stage were collected. Postoperative pathological staging was carried out in accordance with the AJCC 8th version.^13^ The outcomes were defined as follows: disease‐free survival (DFS): the time from the initiation of surgery to disease recurrence or death from any cause; overall survival (OS): the time from the initiation of surgery to death from any cause. The study followed Helsinki declaration, and was approved by the Ethics Committee of the Second Xiangya Hospital of Central South University and patients' informed consent was waived (2023‐015).

### 2.3 Immunohistochemistry (IHC) staining

Four micrometer of paraffin‐embedded tissues were sliced, dewaxed in xylene and hydrated in gradient alcohol. 0.01 M citrate buffer (pH 6.0) and microwave oven were used for heat antigen retrieval. The samples were immersed into a primary antibody of SUZ12 (1:200; Abcam Cambridge, cat. no. ab12073) overnight at 4°C. Next day, the slices were placed into mouse anti‐rabbit polyclonal secondary antibody (working solution, Zhongshan Jinqiao China, cat. no. PV‐9001) for 30 min at 37°C. Two pathology experts assessed staining results through the semiquantitative integral method under double‐blinded conditions. The details were as follows: (1) score for staining intensity: no staining: 0 score; light yellow: 1 score; brown: 2 scores; dark brown: 3 scores; and (2) score for the percentage of positive cells: ≤5%: 0 score; 6%–25%: l score; 26%–50%: 2 scores; 5%–75%: 3 scores; >75%: 4 scores. The product of the above two parameters was the final score, and ≥4 points was classified as high expression.

### 2.4 Cell culture and transfection of lentiviral vectors

A549 (cat. no. SNL‐089), NCI‐H23 (cat. no. SNL‐220), and NCI‐H1650 (cat. no. SNL‐383) LUAD cell lines were purchased from Wuhan Shangen Biotechnology Co., Ltd. DMEM (Sigma–Aldrich, USA; cat. no. D5796) was used to culture A549 cells, and RPMI 1640 medium (Abiowell, China; cat. no. AW‐M002) was used to culture NCI‐H23 and NCI‐H1650 cells. The base medium was added with 10% fetal bovine serum (FBS; Gibco, USA; cat. no. 10099141) and placed at 37°C, 5% CO_2_ cell incubator.

The lentiviral vectors for SUZ12 knockdown (cat. no. GIEL0329222) and overexpression (cat. no. GOSL0345697) were established by Genechem (Shanghai, China). The following target sequences were used for knockdown: sh‐SUZ12‐1, GCTTACGTTTACTGGTTTCTT; sh‐SUZ12‐2, GCTGACAATCAAATGAATCAT; sh‐SUZ12‐3, CCAAACCTCTTGCCACTAGAA; and sh‐NC, TTCTCCGAACGTGTCACGT. The target sequences used for overexpression were as follows: forward, AGGTCGACTCTAGAGGATCCCGCCACCATGGCGCCTCAGAAGCACGG CGGTG; reverse, TCCTTGTAGTCCATACCGAGTTTTTGTTTTTTGCTCTGTTTTG. With the help of polybrene, the cells were transfected with lentiviral vectors, and after transfection for 48 h, subsequent experiments were conducted.

### 2.5 Quantitative real‐time PCR (qRT‐PCR)

TRIzol reagent (Thermo USA, cat. no. 15596026) was applied to extract cell total RNA according to the instructions, then reversely transcribed to cDNA, and the target genes were amplified via a fluorescent quantitative PCR system. The calculation method of 2^−ΔΔCt^ was the ratio of the aim gene expression level in the experimental group to that in the control group. The list of primers sequences: SUZ12 forward, ACATCAAAAGCTTGTCAGCTC; reverse, GCACCTGCTTTTTACCTGTGG; and GAPDH forward, ACAGCCTCAAGATCATCAGC; reverse, GGTCATGAGTCCTTCCACGAT. The other genes' primers sequences were presented in Table S1.

### 2.6 Western blotting

RIPA buffer was applied to extract total protein and quantified with a BCA kit (Abiowell China, cat. no. AWB0104). 10% SDS–PAGE was applied to load and isolate protein specimens. The proteins were transferred to NC membranes, then immersed into the primary antibody diluent overnight at 4°C. Next day, the membrane was cleaned with phosphate‐buffered saline (PBS) and immersed into the secondary antibody at room temperature cultivation for 90 min. The list of primary antibodies: SUZ12 (1 μg/mL, Abcam Cambridge, cat no: ab12073), CDK2 (1:1000; Proteintech, USA, cat. no. 10122‐1‐AP), CDK3 (1:2000; Proteintech, USA, cat. no. 55103‐1‐AP), CDK6 (1:2000; Proteintech, USA, cat. no. 14052‐1‐AP), cyclin D1 (1:5000; Proteintech, USA, cat. no. 26939‐1‐AP), cyclin E1 (1:1000; Proteintech, USA, cat. no. 11554‐1‐AP), p18 (1:1000, BOSTER China, cat. no. M03299‐1), p19 (1:1000, BOSTER China, cat. no. MA1075), p53 (1:5000; Proteintech, USA, cat. no. 60283‐2‐Ig), p‐p53 (1:2000; Proteintech, USA, cat. no. 28961‐1‐AP), p57 (1:1000, BOSTER China, cat. no. BM4129), Rb (1:1000, BOSTER China, cat. no. BM4500), pRb (1:1000, BOSTER China, cat. no. BM4338), Bcl‐2 (1:1000; Proteintech, USA, cat. no. 26593‐1‐AP), Bax (1:2000; Proteintech, USA, cat. no. 50599‐2‐lg), E‐cadherin (1:5000; Proteintech, USA, cat. no. 20874‐1‐AP), N‐cadherin (1:3000; Proteintech, USA, cat. no. 22018‐1‐AP), vimentin (1:4000; Proteintech, USA, cat. no. 10366‐1‐AP), MMP1 (1:1000, BOSTER China, cat. no. A00733‐1), MMP2 (1:500, BOSTER China, cat. no. BM4075), MMP9 (1:1000, BOSTER China, cat. no. PB0709), MMP14 (1:1000, BOSTER China, cat. no. BM4119), TIMP1 (1:1000, Bioss China, cat. no. bs‐0415R), TIMP2 (1:1000, Bioss China, cat. no. bs‐10395R), TIMP3 (1:1000; Proteintech, USA, cat. no. 10858‐1‐AP), ITGB1 (1:1000, BOSTER China, cat. no. BM4308), ITGB3 (1:1000, BOSTER China, cat. no. BA1670), ITGB5 (1:1000, BOSTER China, cat. no. A04201‐1), nm23 (1:1000, BOSTER China, cat. no. BA3787), PD‐L1 (1:3000; Proteintech, USA, cat. no. 66248‐1‐Ig), and β‐actin (1:5000; Proteintech, USA, cat. no. 66009‐1‐Ig). The secondary antibody was goat anti‐mouse/rabbit (1:5000; Abiowell, China, cat. no. AWS0001/cat. no. AWS0002).

### 2.7 Cell proliferation and colony formation assays

Cell Counting Kit 8 (CCK‐8, Dojindo, Japan, cat. no. NU679) experiment was applied to analyze cell proliferation ability. The transfected cells were placed in 96‐well plates (10,000 cells/well) at 37°C, 5% CO_2_ cultivation for 0, 24, 48, or 72 h. Then, the cells were added with 10% CCK‐8 solution (100 μL/well). Microplate reader was used to analyze absorbance values at 450 nm wavelength.

For colony formation assay, six‐well plates were applied to plate cells (400 cells/well) at 37°C, 5% CO_2_ cultivation in 2–3 weeks, until colonies were visible, and then with 4% paraformaldehyde fixed and crystal violet stained the cells, after which the colonies was counted.

### 2.8 Transwell and wound healing assays

For transwell assay, 100 μL of FBS‐free DMEM or RPMI‐1640 with (invasion assay) or without (migration assay) matrigel (200 μg/well, BD, USA, cat. no. 354262) was placed on upper wells of the chamber; the lower wells of chamber were appended with 500 μL whole medium, cultured for 48 h at 37°C, 5% CO_2_ incubator. Next, 4% paraformaldehyde fixed and crystal violet stained the cells; the cells migrated into the lower wells of chamber, then its number was counted.

For wound healing assay, six‐well plates were used to plate cells (5 × 10^5^ cells/well), and lines were drawn with sterilized pipette tips. The debris or detached cells were scoured with PBS, added to FBS‐free medium at 37°C, 5% CO_2_ cultivation for 48 h. Scratches were photographed and the widths were measured at 0 and 48 h.

### 2.9 Cell apoptosis and cycle analyses

Cell apoptosis and cycle were detected by flow cytometry. For cell apoptosis analysis, cells were digested with EDTA‐free pancreatic enzymes, and mixed with 500 μL of binding buffer. Then, 5 μL of Annexin V‐APC (Keygenbio China, cat no: KGA1019) and 5 μL of propidium iodide (PI; MeilunBio China, cat. no. MB2920) were added in turn and mixed. Avoidance of light and reaction at room temperature for 10 min, the samples were analyzed by flow cytometry.

For cell cycle assay, cells were digested with pancreatic enzymes, collected centrifugally, and washed two times with PBS. Three hundred microliters of precooled 100% ethanol was applied to fix cells overnight at 4°C. Collected centrifugally, 150 μL of PI working solution was applied to stain the cells at 4°C for 30 min avoidance of light. In the end, flow cytometry was applied for analysis.

### 2.10 Tumorigenicity in nude mice

Puromycin was used to treat the lentivirus‐infected A549 cells and select stable knockdown SUZ12 cells, and the cells were expanded and collected for qRT‐PCR and western blot analysis.

Four‐week‐old male BALB/c nude mice were purchased from Guangdong Weitong Lihua Laboratory Animal Technology Co., Ltd. Adaptive feed after 1 week, three random groups of nude mice were divided (3 mice/group). A549 cells were inoculated by subcutaneous injection of 2 × 10^6^ cells/100 μL/mouse into the armpit of the anterior right limb. Afterward, observed the growth of the nude mice daily, and tumor measurement was two times a week. The animal experiment was carried out follows the 8th version of NIH guidelines and approved by the Animal Ethics Committee of the Second Xiangya Hospital of Central South University (20220820).

For hematoxylin and eosin (H&E) staining, 4 μm of paraffin‐embedded tissues were sliced, dewaxed and hydrated in turn, and stained with H&E. Pathologists then assessed the sections under a microscope.

For western blot analysis of the animal tissues, 0.025 g of tissue was removed and washed with PBS. After addition of 300 μL of RIPA buffer, the tissue was repeatedly ground until no tissue mass was visible. The remaining procedures of western blot were the same as those in cell experiment.

### 2.11 Chromatin immunoprecipitation (ChIP) assay

The 1.1% formaldehyde solution was applied to cross‐link cells 10 min. The DNA was cut into fragments of 200–1000 bp using an ultrasonic crusher. Protein–DNA complexes were immunoprecipitated by SUZ12 primary antibody (3 μg/200 μL, Abcam Cambridge, cat. no. ab12073), EZH2 primary antibody (3 μg/200 μL, Abcam Cambridge, cat no: ab191250), and H3K27me3 primary antibody (3 μg/200 μL, Abcam Cambridge, cat. no. ab6002) and control IgG antibody (3 μg/200 μL, Abcam Cambridge, cat. no. ab500). After dissociation of the protein–DNA complexes, the DNA fragments were detected via qRT‐PCR; the PCR primers were present in Table S2.

### 2.12 Correlation analysis of SUZ12 and Bax expression

The expression levels of SUZ12 and Bax in paraffin‐embedded tissues from 50 patients with LUAD were detected via IHC. The IHC procedure was the same as that described in Section 2.2. Bax primary antibody (1:200; Proteintech, USA, cat. no. 50599‐2‐lg).

### 2.13 Statistical analysis

The chi‐square test was applied to compare the differences of SUZ12 expression between groups. The Mann–Whitney *U*‐test was applied to compare the differences in ki67 expression between groups. The log‐rank test was applied to test Kaplan–Meier survival curves. The hazard ratios (HRs) was computed through single‐factor Cox proportional hazard model. At least three times were repeated in all cell experiments, two groups of measuring data with two independent samples *t*‐test were compared, and the mean ± standard deviation was applied to express the results. The correlation between SUZ12 and Bax expression was calculated according to Phi correlation coefficient analysis. SPSS 25.0 (IBM, USA) software was applied. Statistical significance was defined by *p* < 0.05.

## 3 RESULTS

### 3.1 SUZ12 was overexpressed in LUAD tissues and correlated with worse clinicopathologic features and poor prognosis

**FIGURE 1:**
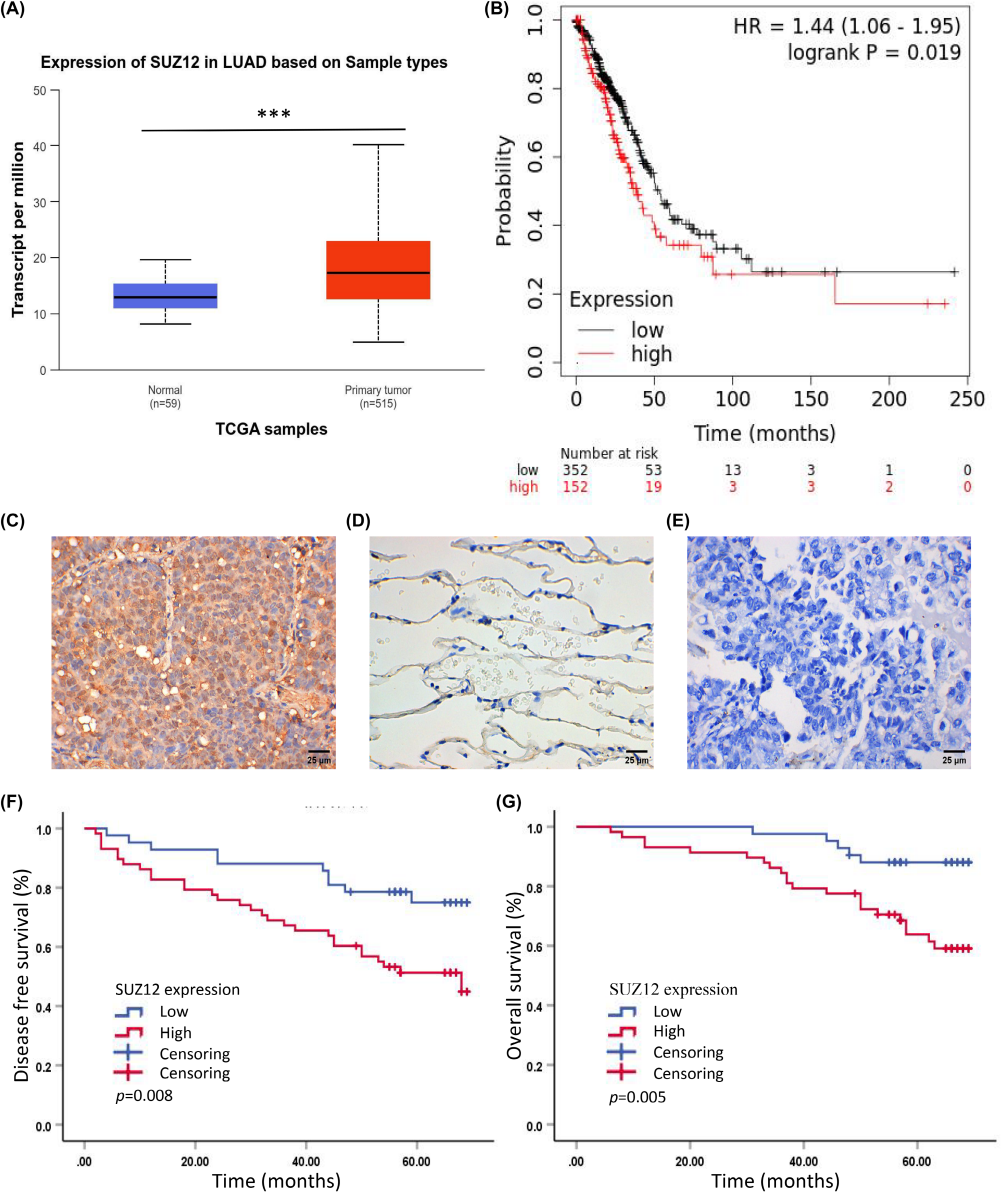
SUZ12 is overexpressed in LUAD tissue and correlated with poor prognosis. The expression of SUZ12 in LUAD tissue and normal tissue was analyzed by UALCAN (A), and OS of LUAD patients with highly or low SUZ12 was analyzed by Kaplan–Meier Plotter (B) online tool from TCGA database. SUZ12 was overexpressed in LUAD tissue (C), negatively expressed in adjacent normal tissue (D) and LUAD tissue with negative control of PBS (E) detected by IHC, × 400. DFS (F) and OS (G) of LUAD patients with highly or low SUZ12 expression was analyzed by log‐rank test. ***p = 1.62E‐12.

First, we investigated SUZ12 expression and prognosis in public database. The bioinformatical analyses results showed that SUZ12 was significantly highly expressed in LUAD tissues than normal tissues (*p* = 1.62E‐12) (Figure 1A), and its high expression was correlated with worse OS from TCGA databases (*p* = 0.019) (Figure 1B).

Next, we tested SUZ12 expression in 100 paraffin‐embedded LUAD tissues and 38 neighboring normal tissues by IHC. There were 44 males and 56 females; the median age was 61 (52–67) years, 4 cases of high differentiation, 18 cases of high to moderate differentiation, 52 cases of moderate differentiation, 20 cases of moderate to poor differentiation, and 6 cases of poor differentiation; and there were 63 cases stage pI disease, 11 cases stage pII disease, 24 cases stage p III disease, and 2 cases stage pIV disease.

SUZ12 protein was positively expressed in the nucleus (Figure 1C–E). The percentage of LUAD tissues with high SUZ12 expression (58.00%, 58/100) was significantly greater than that of neighboring normal tissues with high SUZ12 expression (15.79%, 6/38) (*p* < 0.001) (Table 1). A high expression level of SUZ12 was observed in a large tumor diameter, advanced T stage, lymphatic metastasis, advanced pTNM stage, and poor differentiation groups (*p* < 0.05). Compared with low expression group, Ki67 expression tended to be higher in the SUZ12 high expression group (*p* = 0.144). Moreover, there were no significant correlations between SUZ12 expression and sex, age or smoking status of patients (*p* > 0.05) (Table 1).

**TABLE 1:** Factors were associated with SUZ12 expression.

Variables	SUZ12 expression		*p* value
	Low *n* = 42 (%)	High *n* = 58 (%)	
Sex			
Male	17 (38.6)	27 (61.4)	
Female	25 (44.6)	31 (55.4)	0.546
Age			
<60 years	18 (40.9)	26 (59.1)	
≥60 years	24 (42.9)	32 (57.1)	0.845
Smoking			
No	34 (42.5)	46 (57.5)	
Yes	8 (40.0)	12 (60.0)	0.839
Differentiation			
High/high‐moderate/moderate	35 (47.3)	39 (52.7)	
Moderate‐low/low	7 (26.9)	19 (73.1)	0.070
Diameter			
<3 cm	36 (50.7)	35 (49.3)	
≥3 cm	6 (20.7)	23 (79.3)	0.006
Ki67 (%)	10 (8–30)	20 (10–38)	0.144
pT stage			
pT1	29 (52.7)	26 (47.3)	
pT2‐4	13 (28.9)	32 (71.1)	0.016
pN stage			
pN0	36 (51.4)	34 (48.6)	
pN1‐2	6 (20.0)	24 (80.0)	0.004
pM stage			
pM0	41 (41.8)	57 (58.2)	
pM1	1 (50.0)	1 (50.0)	1.000
pTNM stage			
pI–II	36 (48.6)	38 (51.4)	
pIII–IV	6 (23.1)	20 (76.9)	0.023

By October 2023, the median follow‐up was 62.5 (53.5–67.0) months; there were 39 instances of recurrence and 27 deaths. Compared with the low‐expression group, the SUZ12 high expression group had greater disease recurrence rate (*p* = 0.008) and distant metastasis rate (*p* = 0.016) (Table 2). The median DFS of SUZ12 high expression group was 68 (44–92) months, while that of low‐expression group was not reached (log‐rank test, *p* = 0.008) (HR = 2.543 (1.238–5.225), *p* = 0.011). The median OS was not reached in both the high and low SUZ12 expression groups (log‐rank test, *p* = 0.005) (HR = 3.655 (1.384–9.655), *p* = 0.009) (Figure 1F,G). Among patients followed up for 5 years, compared with the low expression group (82.76%), the SUZ12 high expression group had significantly lower 5‐year OS rate (57.45%) (*p* = 0.023) (Table 3).

**TABLE 2:** Relapse and distant metastasis were associated with SUZ12 expression.

Variables	SUZ12 expression		*p* value
	Low *n* = 42 (%)	High *n* = 58 (%)	
Relapse			
No	32 (76.2)	29 (50.0)	
Yes	10 (23.8)	29 (50.0)	0.008
Distant metastasis			
No	37 (88.1)	39 (67.2)	
Yes	5 (11.9)	19 (32.8)	0.016

**TABLE 3:** Five‐year OS rate was associated with SUZ12 expression.

Variables	SUZ12 expression		*p* value
	Low *n* = 29 (%)	High *n* = 47 (%)	
Survival	24 (82.8)	27 (57.5)	
Death	5 (17.2)	20 (42.5)	0.023

### 3.2 The expression of SUZ12 in LUAD cells

**FIGURE 2:**
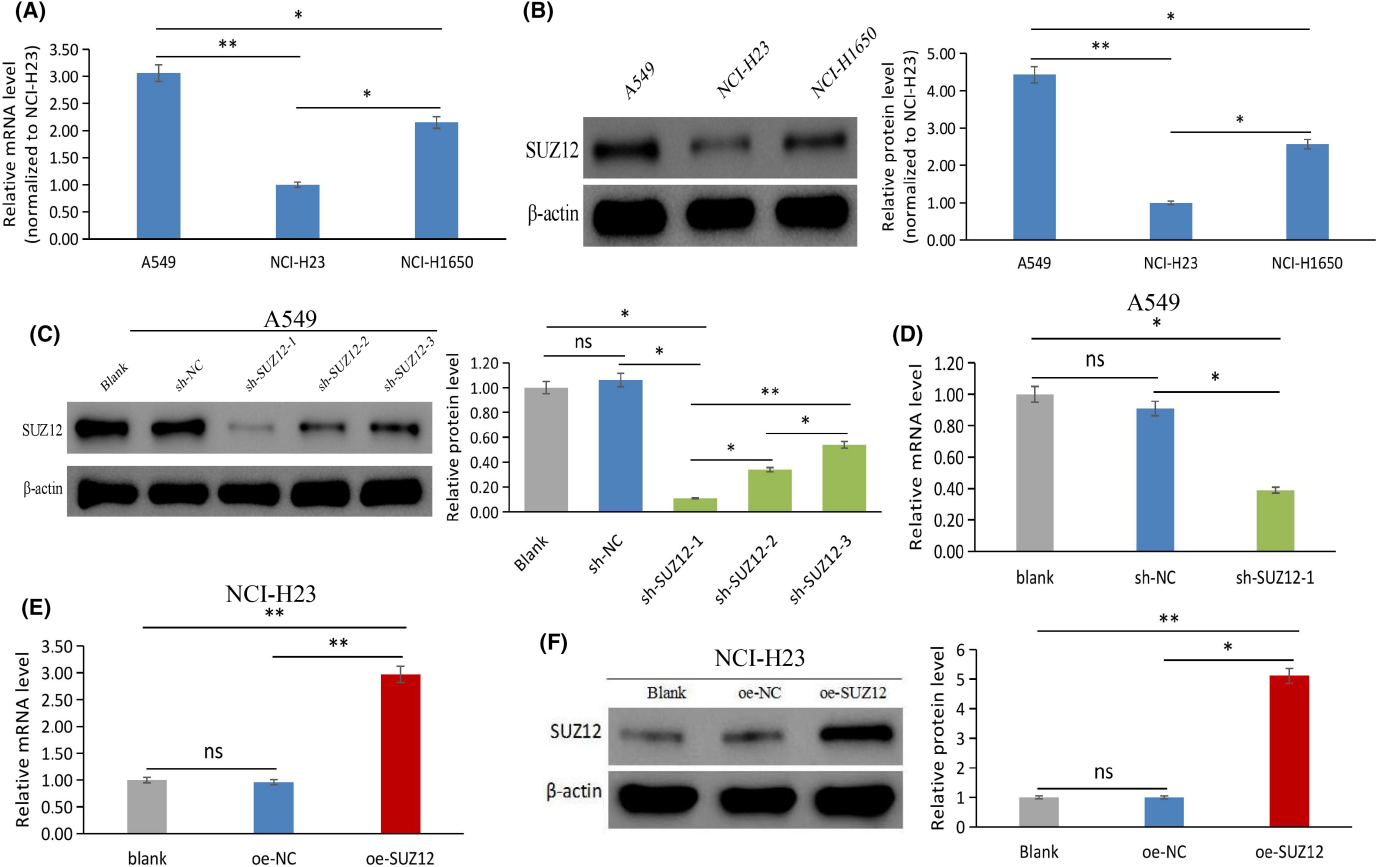
The background expression and lentiviral vector identification of SUZ12 in LUAD cells were tested by qRT‐PCR and western blotting. The expression of SUZ12 mRNA (A) and protein (B) in A549, NCI‐1650, and NCI‐H23 cells lines. The expression of SUZ12 protein (C) and mRNA (D) in sh‐SUZ12‐1/2/3, sh‐NC, and blank groups. The expression of SUZ12 mRNA (E) and protein (F) in oe‐SUZ12, sh‐NC, and blank groups. *p < 0.05, **p < 0.001, ns, no significance.

The expression of SUZ12 in three untreated LUAD cell lines, A549, NCI‐H23, and NCI‐H1650, was measured by qRT‐PCR and western blotting. As a result, both mRNA and protein level of SUZ12 were highly expressed in A549 cells, moderately expressed in NCI‐H1650 cells, and low expressed in NCI‐H23 cells (*p* < 0.05) (Figure 2A,B). Therefore, we chose to knock down SUZ12 in A549 cells and overexpress it in NCI‐H23 cells for the subsequent experiments.

The infection efficiencies of the sh‐SUZ12, oe‐SUZ12, and sh‐NC/oe‐NC lentiviral vectors were all above 98%, as shown in the blank and fluorescent images (Figure S1). The western blotting results revealed that the SUZ12 protein expression of sh‐SUZ12‐1 group was remarkably lower than that sh‐SUZ12‐2, sh‐SUZ12‐3, sh‐NC, and blank groups (*p* < 0.05) (Figure 2C). qRT‐PCR revealed that the SUZ12 mRNA expression of sh‐SUZ12‐1 group was remarkably lower than sh‐NC or blank groups (*p* < 0.05) (Figure 2D). Therefore, we chose sh‐SUZ12‐1 as the shRNA for SUZ12 knockdown in the following experiments. Compared with the oe‐NC or blank groups, oe‐SUZ12 group had obviously elevated the mRNA and protein expression of SUZ12 (*p* < 0.05) (Figure 2E,F).

### 3.3 SUZ12 promotes cell growth, invasion, and migration; inhibits apoptosis; and induces G1/S‐phase cell transition

**FIGURE 3:**
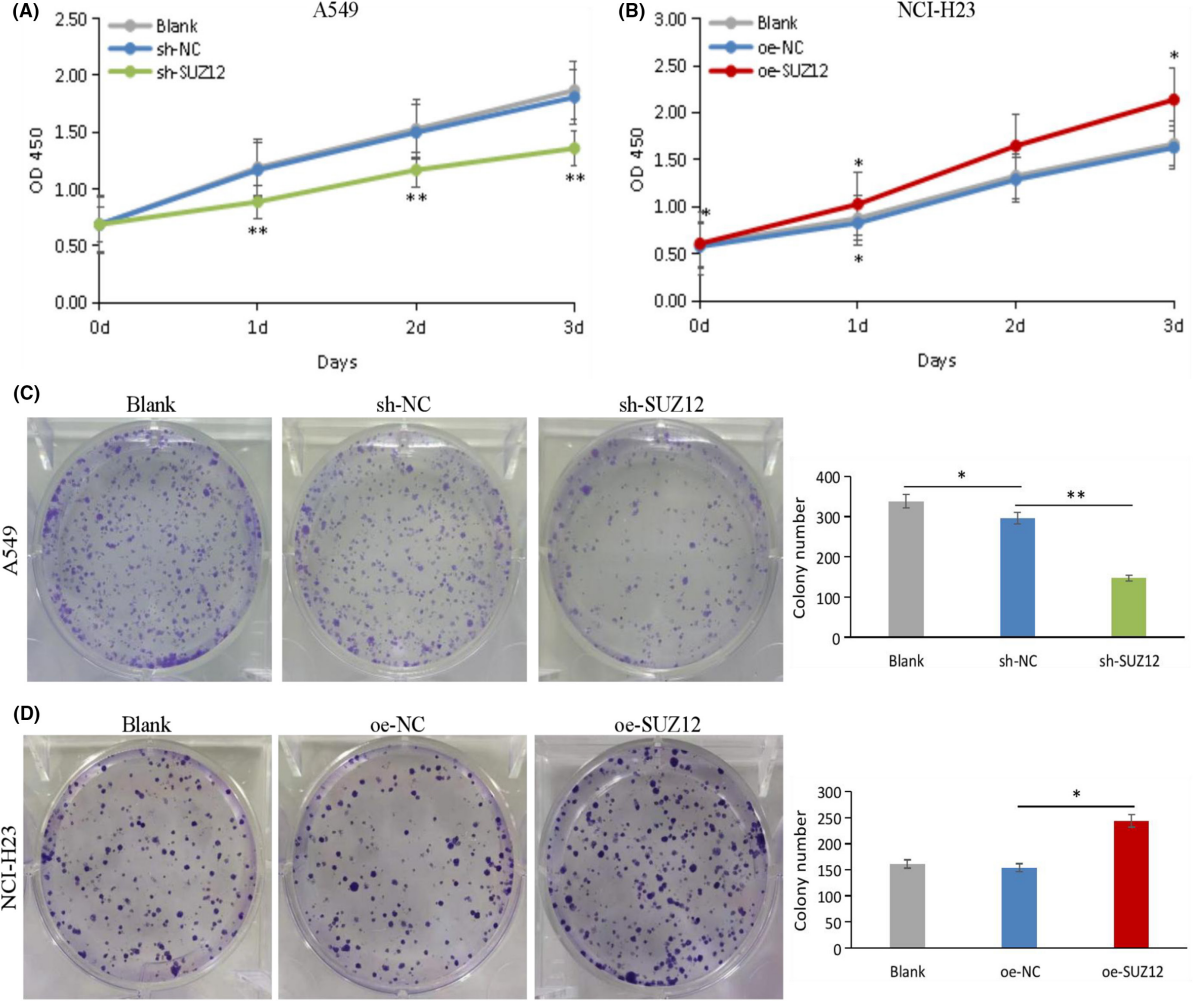
Knockdown of SUZ12 inhibited proliferation and cloning formation in LUAD cells, while overexpression of it displayed opposite effects. Cells proliferation (A, B) and cloning formation (C, D) abilities were tested by CCK‐8 and colony formation assays after knockdown or overexpression SUZ12, respectively. *p < 0.05, **p < 0.001.

**FIGURE 4:**
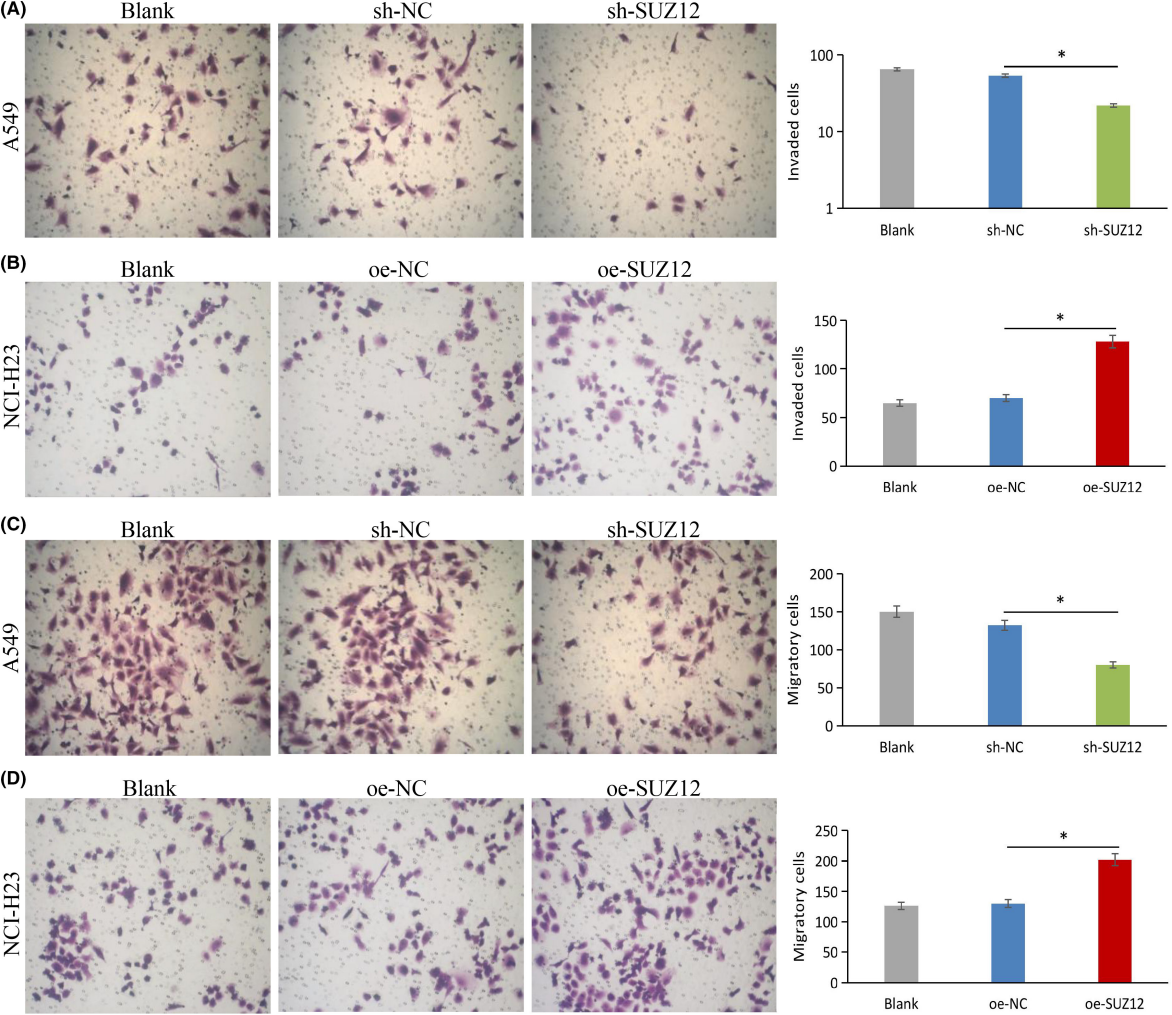
Knockdown of SUZ12 inhibited cell invasion and migration, while overexpression of it displayed opposite effects. Cells invasion (A, B) and migration (C, D) abilities were tested by transwell invasion and migration assays after knockdown or overexpression SUZ12, respectively. *p < 0.05.

**FIGURE 5:**
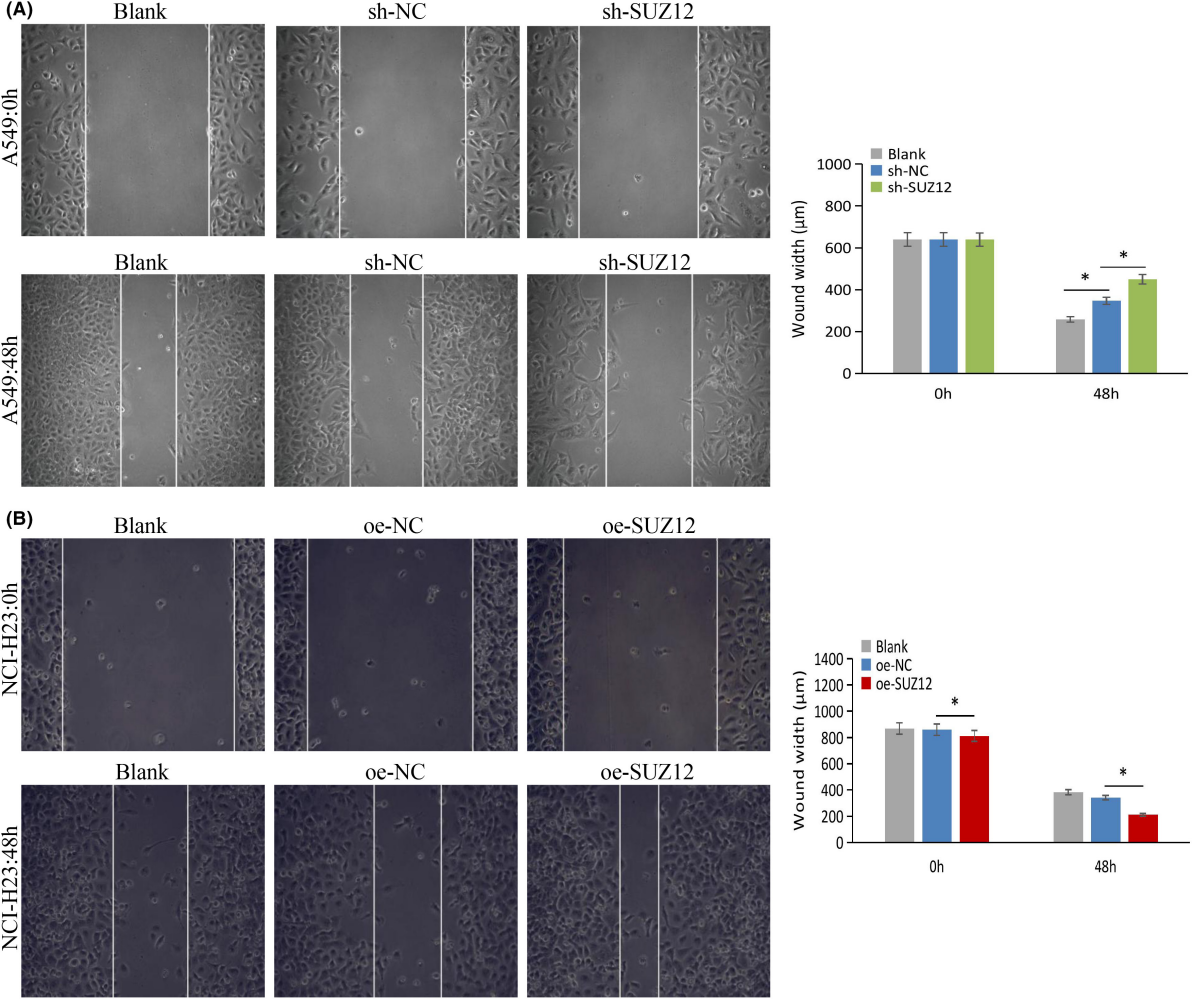
Knockdown of SUZ12 inhibited cell migration, while overexpression of it displayed opposite effects. Cells migration ability (A, B) was tested by wound healing assay after knockdown or overexpression SUZ12, respectively. *p < 0.05.

**FIGURE 6:**
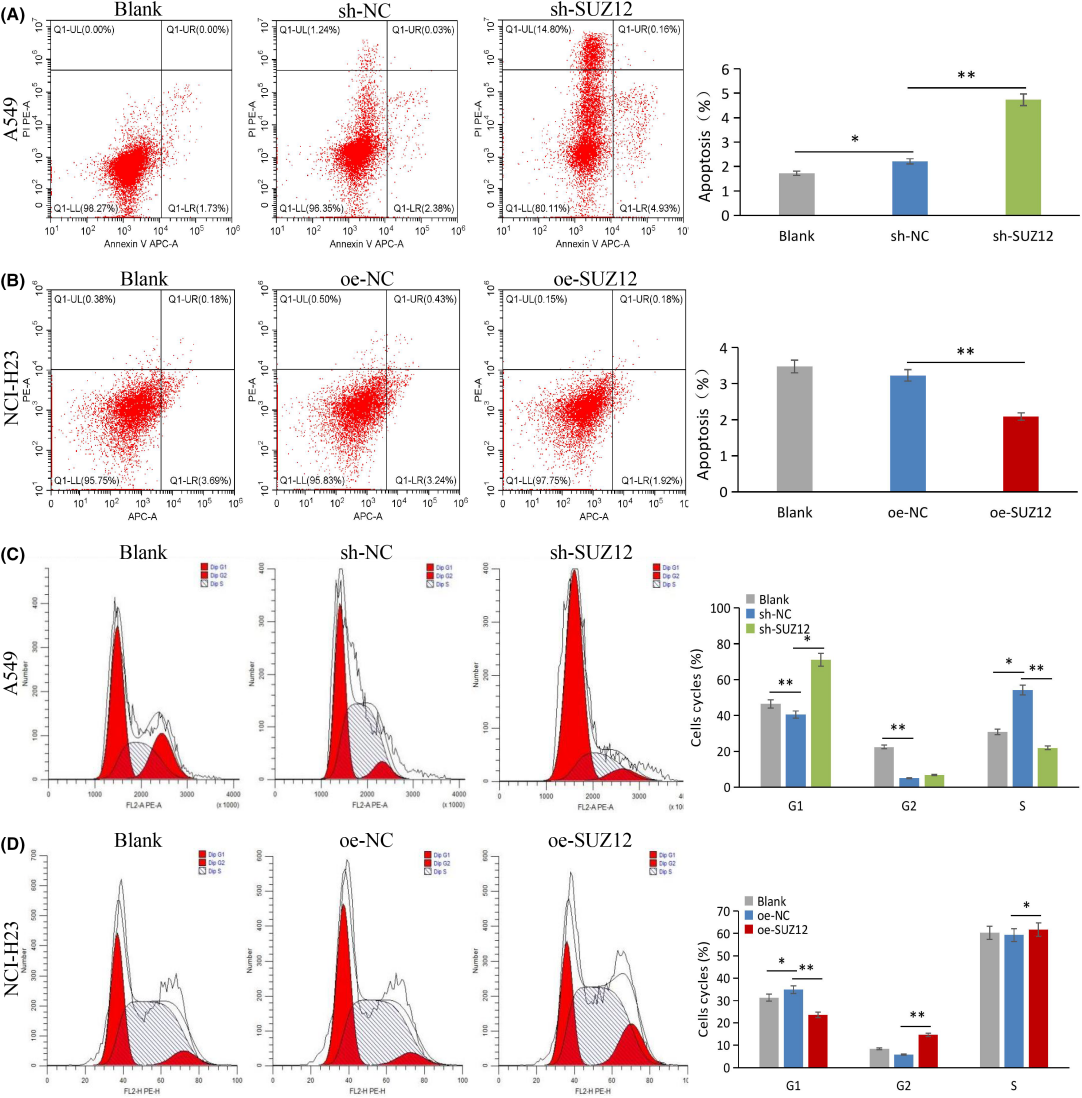
Knockdown of SUZ12 promoted cell apoptosis and G1/S phase arrest, while overexpression of it displayed opposite effects. Cells apoptosis (A, B) and cycle (C, D) were tested by flow cytometry assays after knockdown or overexpression SUZ12, respectively. *p < 0.05, **p < 0.001.

We used a CCK‐8 proliferation assay, a colony formation assay, a transwell invasion/migration assay, a scrape formation assay, and apoptosis/cell cycle flow cytometry assays to examine the phenotype of LUAD cell lines after knockdown or overexpression of SUZ12. As a result, SUZ12 knockdown remarkably inhibited cell proliferation, colony formation, invasion, and migration, and induced apoptosis and G1/S phase arrest (*p* < 0.05), while the opposite effects were observed when SUZ12 overexpression (*p* < 0.05) (Figures 3, 4, 5, 6). In addition, the proportion of G2 phase cells was increased when SUZ12 overexpression (*p* < 0.05) (Figure 6D). The above results indicated that SUZ12 promotes the malignant phenotypes of LUAD cells.

### 3.4 SUZ12 regulates the expression of genes related to cell proliferation and cycle

Cell proliferation and cycle are driven by the core cyclin‐dependent kinases (CDKs)‐cyclins complex. The combination of CDK2/3 with cyclin E^14^ and of CDK4/6 with cyclin D1/2/3^15^ is necessary for the G1/S phase transition. Cyclin‐dependent kinase inhibitor (CKIs), p53 and Rb are key inhibitors of the cell cycle and tumor suppressor genes.

**FIGURE 7:**
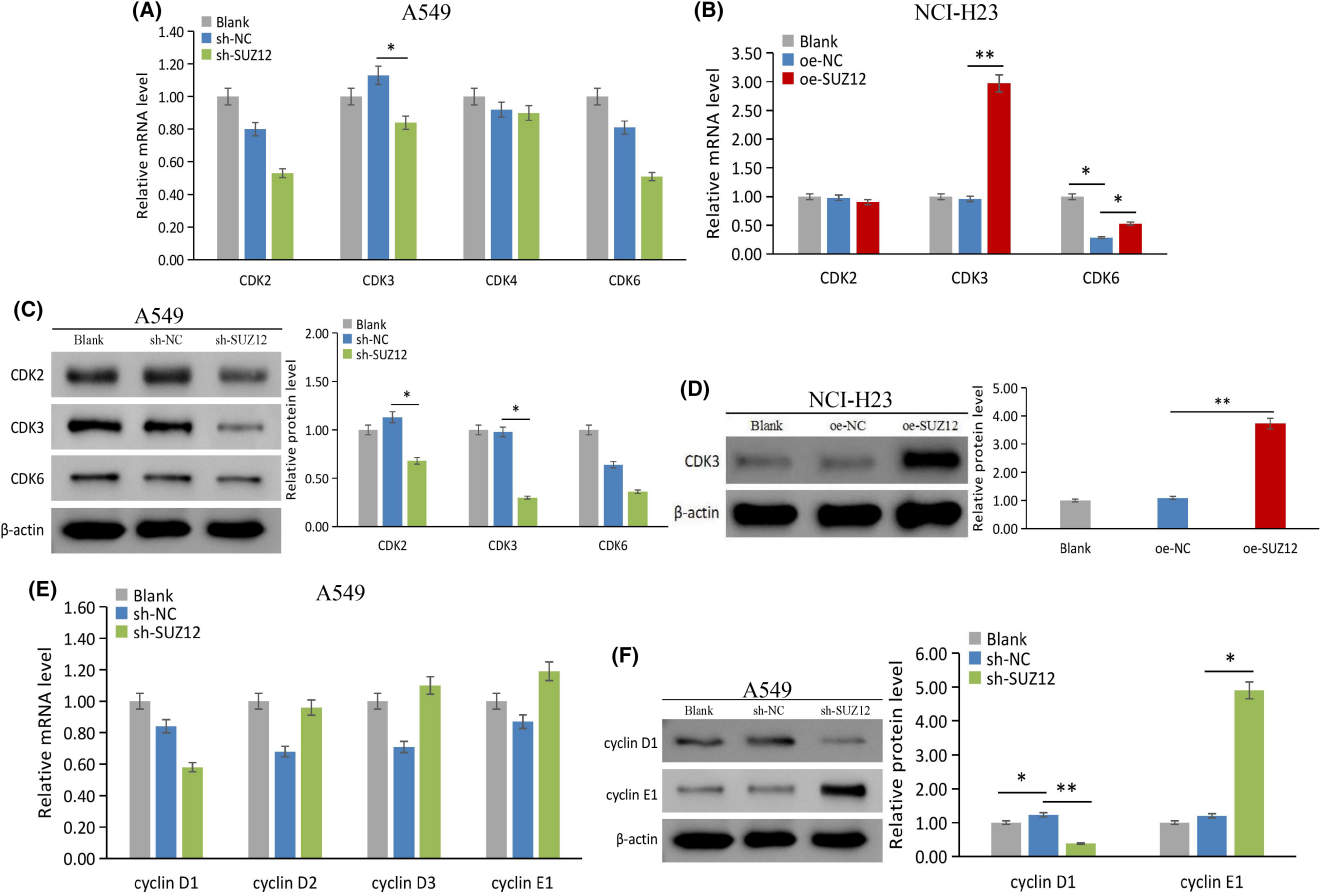
The effect of SUZ12 on CDKs and cyclins expression was tested by qRT‐PCR and western blotting. sh‐SUZ12 decreased CDK3 mRNA (A), CDK2/3 (C), and cyclin D1 (F) protein expression, while increased cyclin E1 protein expression (F), without significantly effected the cyclins mRNA (E). oe‐SUZ12 increased CDK3/6 mRNA (B) and CDK3 protein expression (D). *p < 0.05, **p < 0.001.

The expression levels of CDK2/3/4/6 and cyclin D1/D2/D3/E were detected after knockdown or overexpression of SUZ12. As a result, SUZ12 knockdown significantly decreased the CDK3 mRNA and CDK2/CDK3/cyclin D1 protein levels (*p* < 0.05) (Figure 7). Moreover, overexpression of SUZ12 significantly increased CDK3/CDK6 mRNA and CDK3/cyclin E protein expression (*p* < 0.05) (Figure 7) without changing cyclin mRNA expression (Figure S2A). The above results indicated that SUZ12 may promote cell proliferation and the G1/S phase transition by upregulating CDK2/3 and cyclin D1 expression.

**FIGURE 8:**
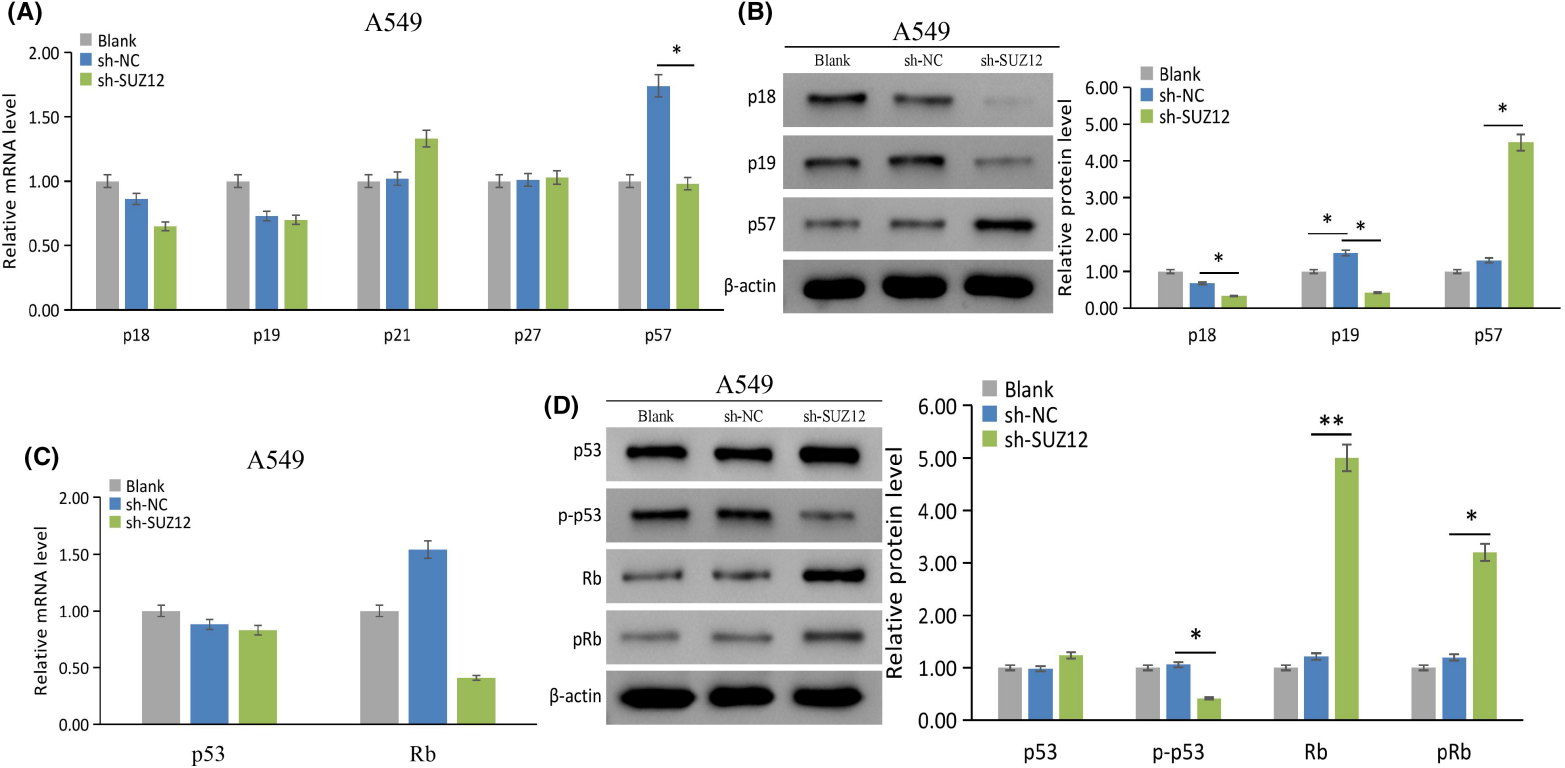
The effect of SUZ12 on CKIs, p53, and Rb expression was tested by qRT‐PCR and western blotting. sh‐SUZ12 decreased p57 mRNA expression (A) and p18/p19/p‐p53 protein expression (B) and (D), while increased p57/Rb/pRb protein expression (B) and (D), without significantly effected the p53 and Rb mRNA expression (C). *p < 0.05, **p < 0.001.

In terms of CKIs, p53 and Rb, SUZ12 knockdown significantly decreased the p57 mRNA and p18/p19/p‐p53 (Ser15) protein levels but increased p57/Rb/pRb (S807) protein levels (*p* < 0.05) (Figure 8). Moreover, SUZ12 overexpression did not obviously change the mRNA levels of CKIs, p53 or Rb (Figure S2B,C). The above results indicated that SUZ12 may promote cell cycle progression through decreasing p57 and Rb protein expression.

### 3.5 SUZ12 upregulates the antiapoptotic factor Bcl‐2 and downregulates the proapoptotic factor Bax

**FIGURE 9:**
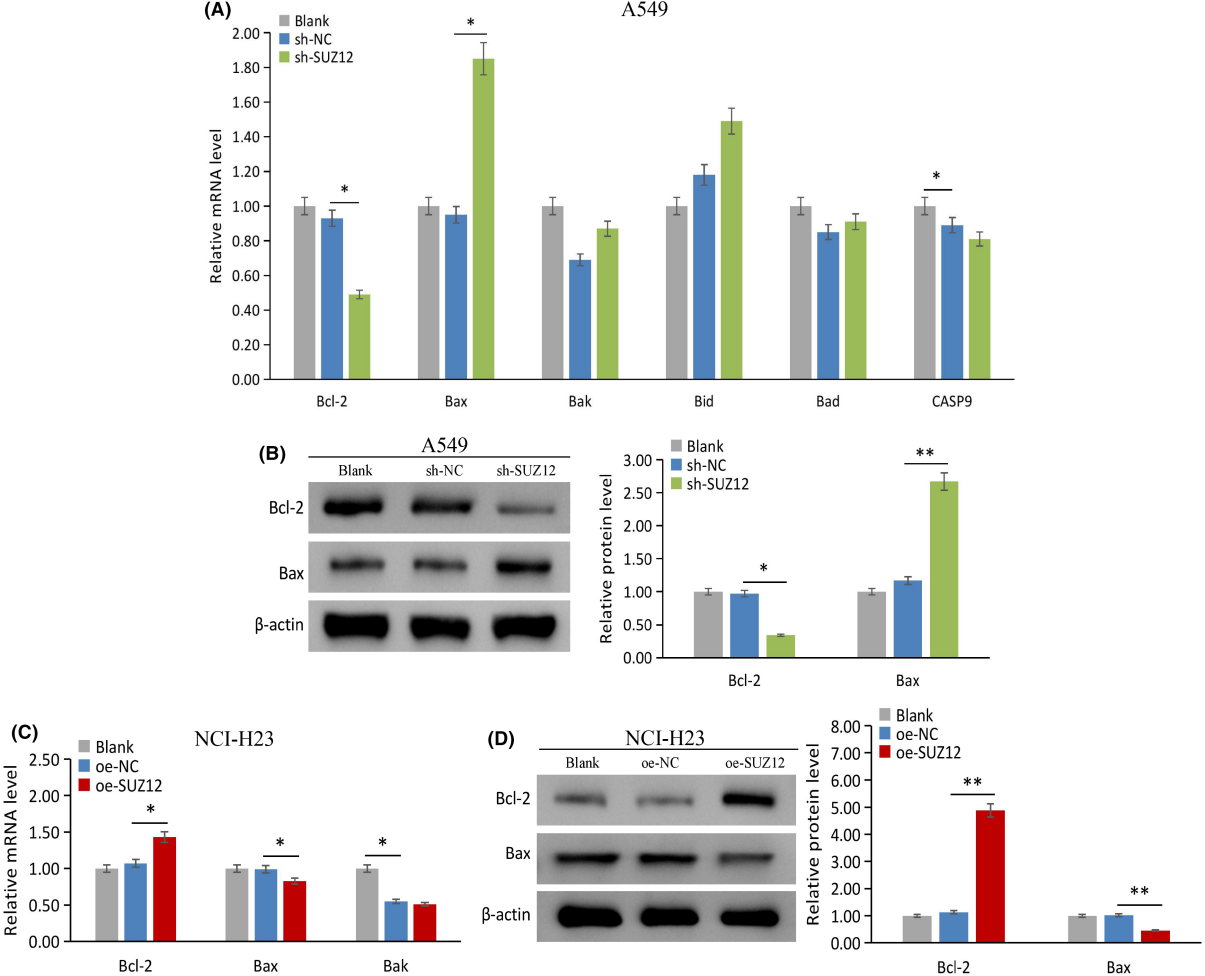
The effect of SUZ12 on the expression of apoptosis‐related genes was tested by qRT‐PCR and western blotting. sh‐SUZ12 decreased Bcl‐2, while increased Bax mRNA and protein expression (A) and (B), oe‐SUZ12 showed the opposite effects (C) and (D). *p < 0.05, **p < 0.001.

Apoptosis plays an important role in the proliferation of tumor. We examined the regulatory effects of SUZ12 on the expression of Bcl‐2, Bax, Bak, Bid, Bad, and CASP9, which are important apoptotic regulators. The mRNA and protein level of bcl‐2 was significantly reduced, while Bax was significantly increased (*p* < 0.05), when SUZ12 knockdown (Figure 9). The opposite effects were observed when SUZ12 overexpression (*p* < 0.05) (Figure 9). The above results indicated that SUZ12 may upregulate Bcl‐2 and downregulate Bax expression to play an antiapoptotic role.

### 3.6 SUZ12 regulates the expression of genes related to cell migration

**FIGURE 10:**
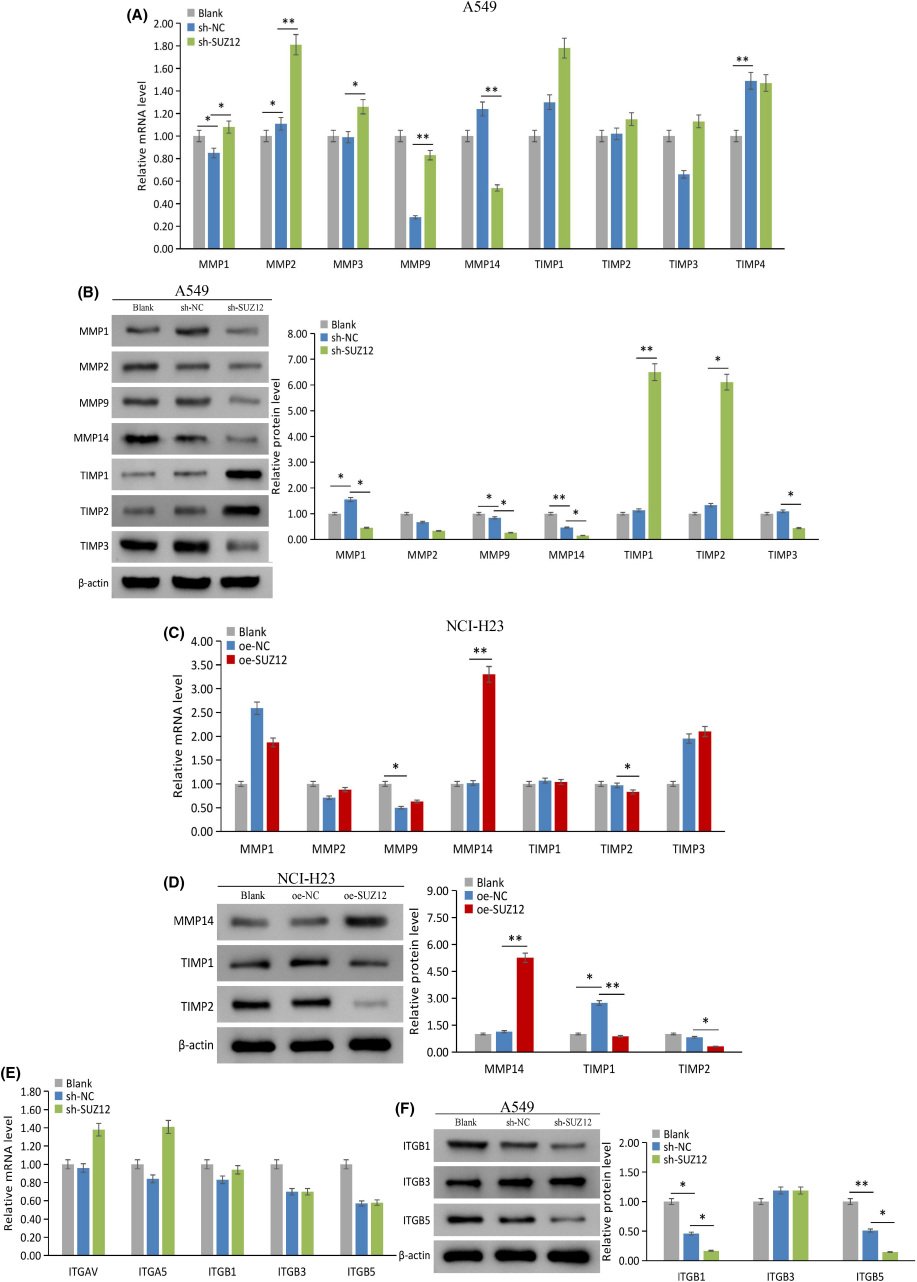
The effect of SUZ12 on metastasis related genes expression was tested by qRT‐PCR and western blotting. sh‐SUZ12 increased MMP1/2/3/9 mRNA expression (A) and TIMP1/2 protein expression (B), while decreased MMP14 mRNA expression (A), MMP1/9/14 (B), TIMP3 (B), and ITGB1/5(F) protein expression, without significantly effected ITGBs mRNA expression (E). oe‐SUZ12 increased MMP14 mRNA (C) and protein (D) expression, while decreased TIMP2 mRNA expression (C) and TIMP1/2 (D) protein expression.

The expression of matrix metalloproteinases (MMPs)/tissue inhibitors of metalloproteinases (TIMPs), epithelial‐to‐mesenchymal transition (EMT), integrins, and nm23 was detected after knockdown or overexpression of SUZ12. As a result, SUZ12 knockdown significantly increased MMP1/2/3/9 mRNA levels and TIMP1/2 protein levels, but decreased MMP14 mRNA levels and MMP1/9/14, TIMP3, and ITGB1/5 (Figure 10) protein levels (*p* < 0.05); there was a marginal decrease in MMP2 protein expression (*p* = 0.051) (Figure 10). Overexpression of SUZ12 significantly increased MMP14 mRNA and protein level (*p* < 0.001), but reduced TIMP2 mRNA and TIMP1/2 protein level (*p* < 0.05) (Figure 10).

In terms of EMT, SUZ12 knockdown significantly decreased E‐cadherin mRNA (*p* = 0.008) and E‐cadherin protein levels (*p* < 0.05) but increased vimentin protein expression (*p* = 0.004), with no significant effects on the mRNA or protein expression of other EMT markers (Figure S3) or nm23 (Figure S3). Consistently, overexpression of SUZ12 significantly increased E‐cadherin mRNA expression (*p* = 0.005) (Figure S3).

These results suggested that SUZ12 may upregulate MMP1/2/9/14 and ITGB1/5 and downregulate TIMP1/2 protein expression to promote tumor invasion and metastasis. However, EMT may not be the main mechanism regulated by SUZ12 in A549 cells.

### 3.7 SUZ12 regulates the expression of the immunity‐related factor PD‐L1

**FIGURE 11:**
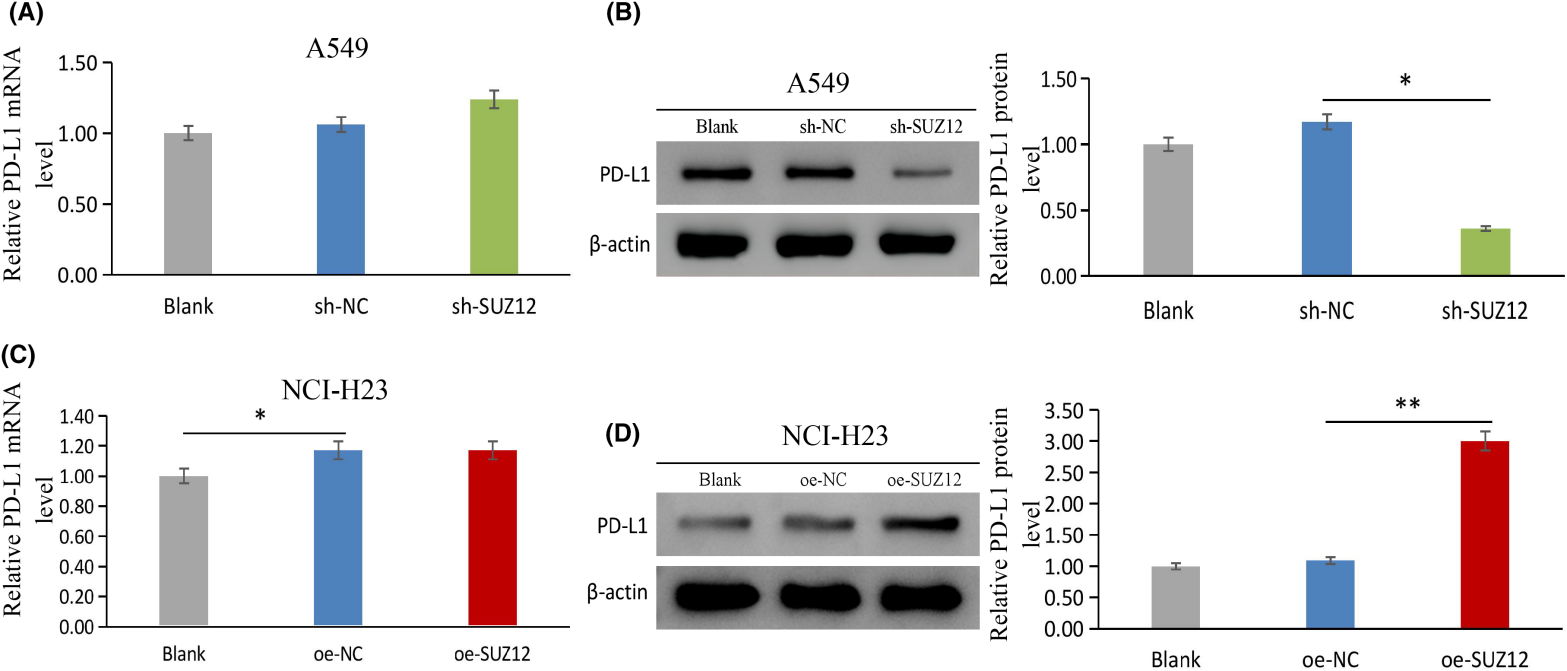
The effect of SUZ12 on PD‐L1 expression was tested by qRT‐PCR and western blotting. sh‐SUZ12 (A) and oe‐SUZ12 (C) had no significant effect on PD‐L1 mRNA expression. sh‐SUZ12 reduced PD‐L1 protein expression (B), while oe‐SUZ12 showed the opposite effect (D).*p < 0.05, **p < 0.001.

Finally, we evaluated whether SUZ12 regulates the key immune molecule PD‐L1. The results displayed that PD‐L1 protein level was obviously decreased by SUZ12 knockdown (*p* = 0.008), while the opposite effect was observed for SUZ12 overexpression (*p* < 0.001) (Figure 11). These results indicated that SUZ12 may up‐regulate PD‐L1 protein expression to participate in tumor immunosuppression.

### 3.8 Knockdown of SUZ12 reduced the tumorigenicity of LUAD cells and regulated the expression of Bcl‐2 and Bax in vivo

**FIGURE 12:**
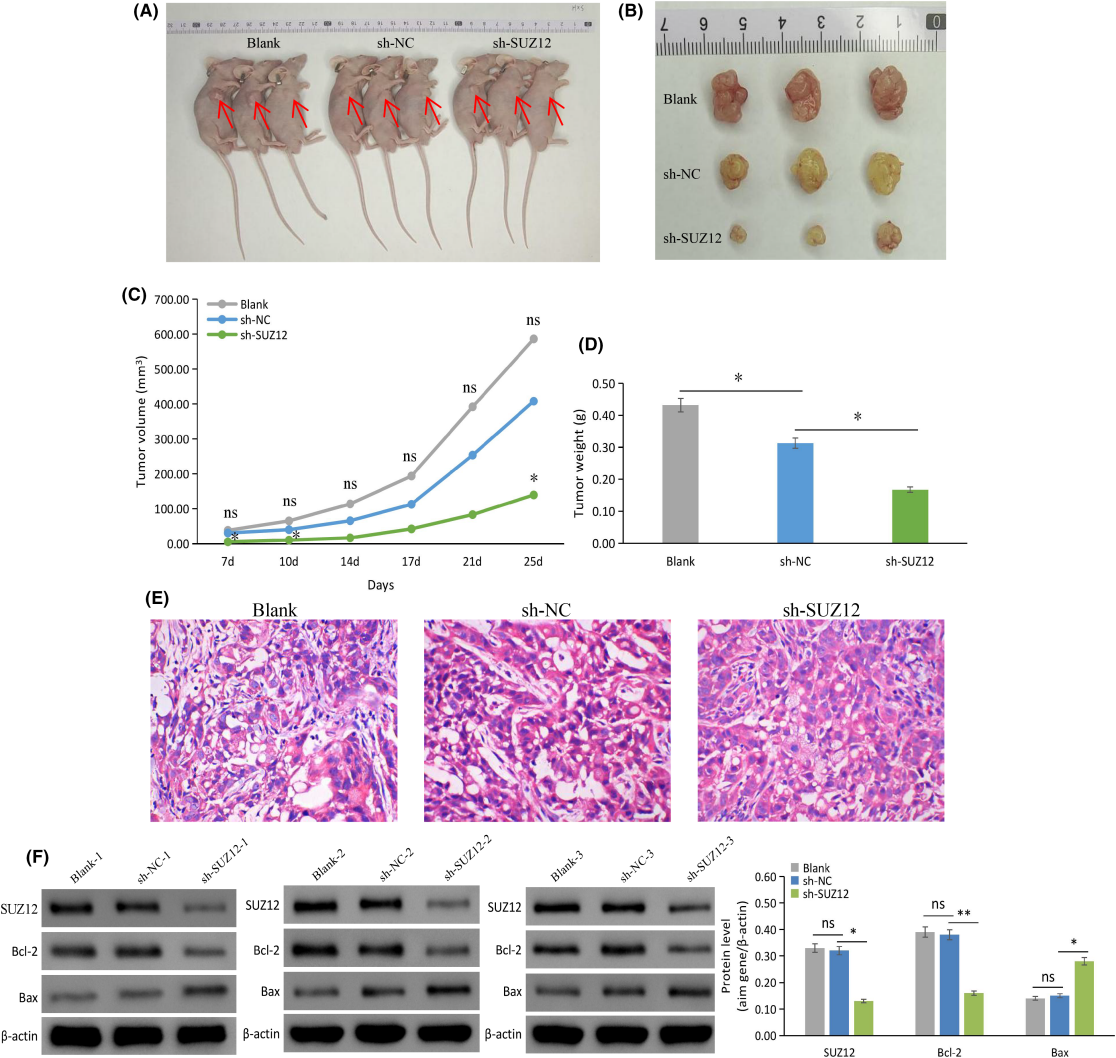
Knockdown SUZ12 reduced tumorigenicity and regulated apoptosis‐related genes expression in nude mice. The overall picture of nude mice without tumor removal (A), subcutaneous tumor of nude mice after tumor removal (B), the growth of tumor volume in nude mice in sh‐SUZ12, sh‐NC and blank groups (C), the weight of tumor from nude mice in sh‐SUZ12, sh‐NC, and blank groups (D), H&E staining of tumor tissue in nude mice (E), × 400, western blot analysis of SUZ12, Bcl‐2, and Bax expression in sh‐SUZ12, sh‐NC and blank groups of tumor from nude mice (F). *p < 0.05, **p < 0.001, ns, no significance.

We successfully constructed stable sh‐SUZ12 and sh‐NC‐transfected A549 cells (Figure S4), and utilized a xenograft model of nude mice after subcutaneous injection with above stable and blank A549 cells (Figure 12A). Compared with sh‐NC, sh‐SUZ12 noticeable suppressed growth of tumor in gross specimens, and significantly decreased the tumor volume on day 7 (*p* = 0.041), day 10 (*p* = 0.031), and day 25 (*p* = 0.037) and decreased the tumor weight on day 25 (*p* = 0.047) (Figure 12B–D).

H&E staining confirmed that all the tumor tissues removed from the mice were cancerous (Figure 12E). The western blotting results displayed that the sh‐SUZ12 group had significantly lower SUZ12 protein expression than the sh‐NC group (*p* = 0.001), indicating that the tumorigenicity model of SUZ12 knockdown was successfully established in the nude mice (Figure 12F).

Based on the above results, SUZ12 effectively regulated the mRNA and protein expression of Bcl‐2 and Bax. Therefore, we chose Bcl‐2 and Bax as target molecules for the in vivo study. Western blotting results showed that sh‐SUZ12 group had significantly lower protein expression of Bcl‐2 (*p* < 0.001), but had significantly higher Bax protein expression (*p* = 0.005), compared with sh‐NC group (Figure 12F). These results indicated that SUZ12 knockdown inhibits the tumorigenicity of A549 cells and regulates the expression of Bcl‐2 and Bax in vivo.

### 3.9 SUZ12 can bind to the promoter region of Bax

Owing to the direct mechanism by which SUZ12 silences tumor suppressor genes, we chose Bax as a promoter target gene. Through the use of the hTFtarget human transcription factor database (http://bioinfo.life.hust.edu.cn/hTFtarget#!/prediction), we found that there were multiple SUZ12 binding sites in the Bax promoter (all *p* values<5E‐05). Thus, we considered these potential binding sites to be highly reliable, and four of them were chosen as validation sites (Table 4).

**TABLE 4:** SUZ12 and EZH predicted binding sites on the Bax gene promoter.

Genes	Strand (+/−)	Binding sites (start/stop)	Predicted matched core sequences
SUZ12			
Binding 1	+	254/269	CCCCTGCCTCAGCTCC
Binding 2	−	−1095/−1079	CTCCTGCCTCAGCCTC
Binding 3	+	1121/1136	TGTGGCCCCTCCCCTC
Binding 4	−	−42/−26	CGCCGCCGGGTCACGT
EZH2			
Binding 1	+	73/94	TGAAATTAAAACACTGCACACA
Binding 2	−	−1857/−1835	TCAAAAAAAAAAAAAAAAAAAA
Binding 3	−	−1376/−1354	AGAAAATAATAGCAACTAAGGA
Binding 4	+	1501/1522	TCAAAAAAAAAAAAAAAAAAAA

In addition, we aimed to verify whether EZH2 and H3K27me3 also have binding sites in the Bax promoter and whether they depend each other as transcription factors. Through the hTFtarget database, we also found multiple EZH2 binding sites in the Bax promoter (all *p* values<9E‐05). We chose four prediction sites with high confidence as verification sites (Table 4) (common primers for the 1st and 2nd sites because the complementary sequence of the 2nd site on the positive chain was very close to the 1st site). Since H3K27me3 was not included in the transcription factor database, we found primer sequences that bind to the Bax promoter in literature^16^ (Table 4).

**FIGURE 13:**
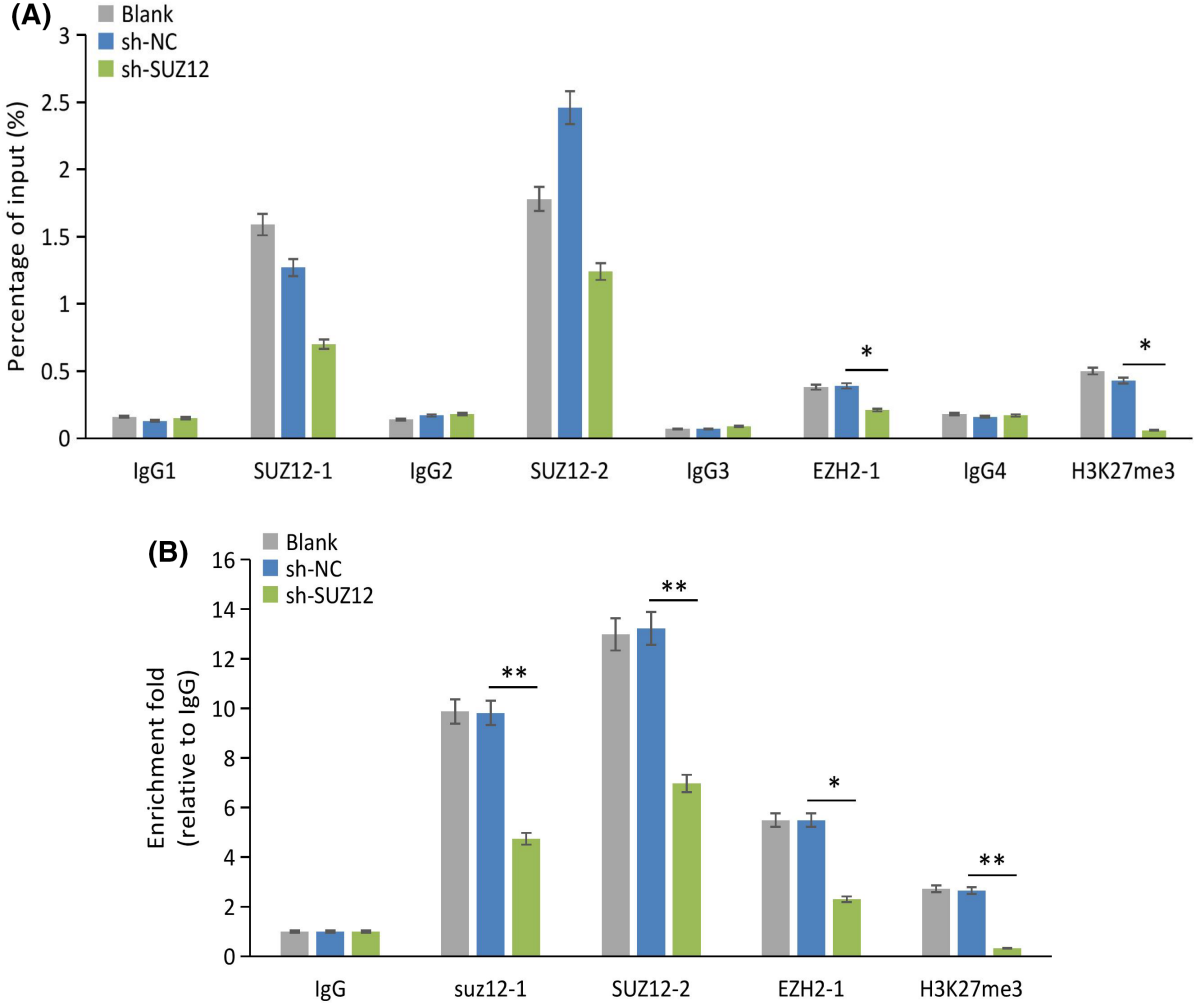
The binding of SUZ12 to Bax promoter region was tested by ChIP. The proportion of amount of DNA fragments (Input %) corresponding to prediction sites on Bax promoters pulled down by each indicator (A). The enrichment fold analysis of SUZ12, EZH2 and H3K27me3 at Bax promoter region (B). SUZ12‐1 and SUZ12‐2: SUZ12 prediction sites 1 and 2 at Bax promoter region, EZH2‐1: EZH2 prediction sites 1 and 2 (common primers) at Bax promoter region. *p < 0.05, **p < 0.001.

In the ChIP–PCR assay, the PCR dissolution peaks of the SUZ12 primer 1/2, EZH2 primer 1, and H3K27me3 primer were unimodal (Figure S5A,B,E,H), but the SUZ12 primers 3/4 and EZH2 primers 2/3 were bimodal (Figure S5C,D,F and G), so we only retained unimodal primers in following experiments. Quantitative analysis of the PCR products revealed that SUZ12 could specifically bind to Bax promoter prediction sites 1/2, EZH2 could bind to prediction sites 1/2, and H3K27me3 could bind to the predicted site (Figure 13A). The enrichment results showed that SUZ12 prediction site 2 had the highest enrichment in the Bax promoter, followed by SUZ12 prediction site 1, EZH2 prediction sites 1/2 (common primers), and the H3K27me3 binding site. Knockdown of SUZ12 significantly reduced the enrichment of SUZ12 at prediction sites 1/2 (both *p* < 0.001) and also significantly reduced the enrichment of EZH2 (*p* = 0.002) and H3K27me3 (*p* < 0.001) at the Bax promoter (Figure 13B).

The above results suggested that SUZ12 can bind to the Bax promoter (254/269 bp and − 1095/−1079 bp) and EZH2 and H3K27me3 levels dependents on SUZ12.

### 3.10 SUZ12 expression is negatively associated with Bax expression in LUAD tissues

Finally, the correlation between SUZ12 and Bax protein expression was analyzed. A total of 50 paraffin‐embedded human LUAD tissues were included for analysis, and the corresponding patients included 23 males and 27 females, with a median age 61 (50–67) years old, 1 case of high differentiation, 10 cases of high to moderate differentiation, 28 cases of moderate differentiation, 7 cases of moderate to poor differentiation, 4 cases of poor differentiation, 33 cases of pI stage disease, 6 cases of pII stage disease, and 11 cases of pIII stage disease.

**FIGURE 14:**
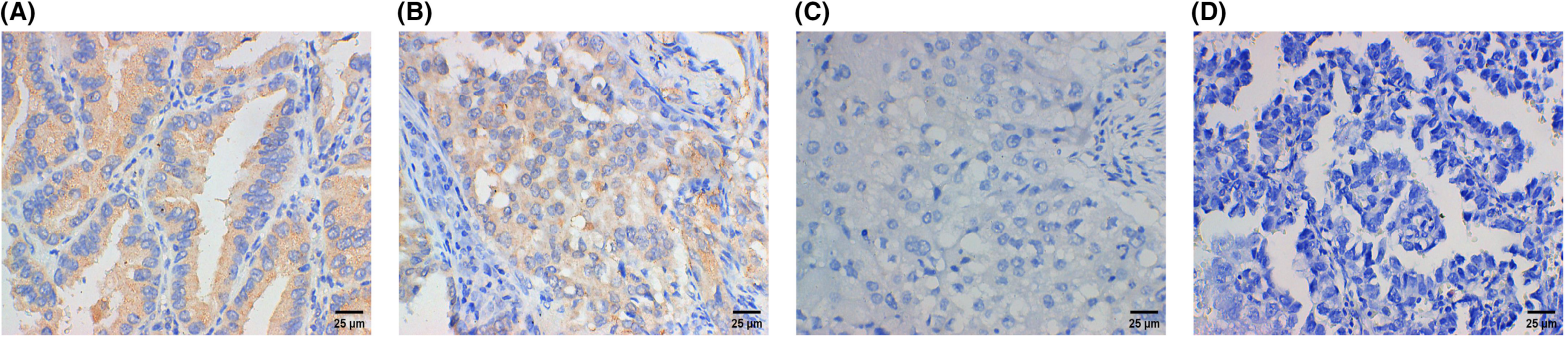
The expression of Bax in human LUAD tissue was detected by IHC. High expression (A), low expression (B), negative expression (C), and negative expression in the negative control of PBS (D). × 400.

Bax was detected in the cytoplasm (Figure 14) by IHC, and a high expression rate was detected for 46% (23/50). We selected 50 samples (out of 100 samples from the above results of SUZ12 IHC staining) that paired with Bax for analysis of SUZ12 expression; the percentage of samples with high SUZ12 expression was 62% (31/50). As a result, the protein expression of SUZ12 and Bax were negatively correlated (*R* = −0.352, *p* = 0.013) (Table 5).

**TABLE 5:** Correlation of SUZ12 and Bax expressions in human lung adenocarcinoma tissue.

Variables	SUZ12 low expression *n* = 19 (%)	SUZ12 high expression *n* = 31 (%)	Phi R	*p* value
Bax low expression *n* = 27 (%)	6 (22.2)	21 (77.8)		
Bax high expression *n* = 23 (%)	13 (56.5)	10 (43.5)	−0.352	0.013

## 4 DISCUSSION

SUZ12 was reported to be overexpressed in tissues of some cancer types and its upregulation is correlated with worse clinicopathological features or a poor prognosis. High SUZ12 protein expression was detected in 55.72% of HNSCC (112/201), but only in 15% (3/20) of oral mucosal tissues (*p* < 0.0001), and this phenotype was associated with lymphatic metastasis, shorter DFS and shorter OS.^6^ High SUZ12 protein expression was detected in 76.9% of ovarian cancer tissues (90/117), and this rate was higher than the high expression rate in the normal ovarian epithelium (20.0%, 7/35) and fallopian tube epithelium (13.3%, 2/15); this phenotype was also correlated with poor OS.^7^ SUZ12 is also overexpressed at the mRNA level in gastric cancer^8^ and colon cancer^17^ tissues, and it associated with tumor diameter, lymphatic metastasis, stage and prognosis. Martín‐Pérez et al.^18^ reported that the percentage of SUZ12‐positive tumors in tumor tissue chips was 50.3% (250/497), and its expression level was higher in highly aggressive tumors. However, the SUZ12 protein is expressed at low levels in HBV‐associated liver cancer tissues, and improved prognosis was observed in high expression group.^19^ Therefore, the above results suggested that SUZ12 expression and its significance may be related to tumor type. In NSCLC, compared with neighboring normal tissues, SUZ12 mRNA expression was higher in 40 cancer tissues and associated with worse clinicopathological features.^9^ However, there are no related reports on the protein expression and prognostic implications of SUZ12 in LUAD. Our study showed that SUZ12 protein was expressed at a higher level in LUAD tissues than in neighboring normal tissues and associated with tumor size, lymphatic metastasis, and stage, which was in accord with the previous studies' findings. Poor prognosis was observed in SUZ12 high expression group, especially the 5‐year OS rate, which suggests that it may be a valuable prognostic marker and potential therapeutic target for LUAD.

Studies reported that SUZ12 knockdown inhibited the proliferation, colony formation, invasion and migration of HNSCC and gastric cancer cells.^6^, ^8^ SUZ12 was shown to be necessary for the malignant transformation of cells induced by arsenic trioxide in a mouse model.^20^ Knockdown of SUZ12 also induces cell apoptosis in gastric cancer,^8^ ovarian cancer,^7^ and mantle cell lymphoma cells^18^ and induces G1/S phase arrest in gastric cancer cells.^10^ In our study, knockdown of SUZ12 inhibited the proliferation, colony formation, invasion and migration of LUAD cells and promoted apoptosis and G1/S phase cycle arrest, while overexpression of SUZ12 had the opposite effects. Therefore, we confirmed that SUZ12 plays a procancer role in LUAD cells through both knockdown and overexpression experiments. In addition, our study suggested that SUZ12 may promote G2 phase process.

At present, regarding the roles of molecular mechanism of SUZ12 in promoting cancer, previous studies displayed that it may silence p21^11^ and p27^12^ and upregulate cyclin D1^6^ and MMP2/9^21^ expression. However, other related mechanisms remain unclear, such as on proliferation, invasion, metastasis, apoptosis, and immunity of tumor. In present study, we systematically explored the impact of SUZ12 on the above key signaling molecules. The results showed many key molecules are regulated by SUZ12. Among them, as far as our best knowledge, we first reported CDK3, p57, Bcl‐2, Bax, MMP1/14, TIMP2, ITGB1/5, and PD‐L1 were regulated by SUZ12, and SUZ12 can directly bind to Bax promoter.

SUZ12 may upregulate CDK2/3 and cyclin D1 and downregulate p57 and Rb to promote cell cycle progression. Among them, SUZ12 positively regulated CDK2/3 (especially CDK3) and cyclin D1 expression, which were related to promote cell G1/S transition. p57, a new member of the Kip/Cip family, is a broad‐acting CKI that is expressed in G1 and S phases of cells, inhibits DNA replication, and negatively regulates the cell cycle, its main substrate is CDK2, and it deletion or inactivation is common in human lung cancer cells.^14^ p53 and Rb are two key suppressors of cancer and the cell cycle. This study showed that SUZ12 knockdown increased the total protein level of Rb but not the total protein level of p53. Interestingly, we found that SUZ12 knockdown decreased p‐p53 and increased pRb protein levels, which indicated that SUZ12 may simultaneously induce procancer and anticancer signaling pathways. However, further, we found that the fold change in Rb expression (3.09 times) was greater than that of pRb (1.29 times) following the knockdown of SUZ12, which indicated that SUZ12 mainly exerts a procancer effect.

MMPs/TIMPs, EMT‐related factors and integrins are key regulatory factors involved in tumor invasion and metastasis.^4^, ^22^ Our results showed that SUZ12 may upregulate MMP1/2/9/14 and ITGB1/5 and downregulate TIMP1/2 protein expression to promote invasion and migration. In retinoblastoma, knockdown of SUZ12 reduced MMP2/9 protein expression.^21^ There are no related reports of the regulation of integrin by SUZ12. However, studies have shown that SUZ12 promotes the EMT process in gastric cancer and bladder cancer cells.^8^, ^23^ However, we did not find similar results in LUAD cells, except for the N‐cadherin marker. We speculated that this difference may be related to the cell type.

Cancer cells can evade immune surveillance by expressing immunosuppressive proteins, for example PD‐L1. SUZ12 was reported to participate in tumor immunosuppression in previous studies,^24^, ^25^ but there are no related reports of its relationship with PD‐L1. Our study showed that SUZ12 led to marked upregulation of PD‐L1 protein expression, which may be related to tumor immune escape.

Apoptosis plays an important role in cell proliferation. As discussed above, our study showed that SUZ12 effectively inhibited cell apoptosis. Mechanistically, we found that SUZ12 upregulated Bcl‐2 but downregulated Bax in vitro and vivo. The Bcl‐2 family is a famous regulator of apoptosis and includes antiapoptotic proteins (Bcl‐2, Bcl‐xL, etc.) and proapoptotic proteins (Bax, Bak, etc.). In the case of cell homeostasis, Bcl‐2 can directly bind to Bax/Bak and inhibit their activity.^14^ However, under cellular stress, the inhibitory effect of Bcl‐2 on Bax/Bak was relieved, resulting in oligomerization of the latter two proteins and the formation of mitochondrial membrane permeability transport pores, then the cytochrome C was released into the cytoplasm and induced apoptosis, which is the main pathway involved in endogenous apoptosis.^22^ Owing to the direct effect of SUZ12 on silencing the expression of tumor suppressor genes, we chose Bax as a target promoter study of SUZ12. Through the ChIP assay, SUZ12 was shown to directly bind to the Bax promoter region, EZH2 and H3K27me3 levels dependents on SUZ12. EZH2 and SUZ12 are two core members of PRC2, this complex can trimethylates H3K27 to form H3K27me3, the latter tightens chromatin and blocks gene transcription,^5^ which is one of the two main transcriptional silencing mechanisms in mammals.^4^ Therefore, our study showed that SUZ12 acts as a transcription factor by binding to the Bax promoter in the form of the PRC2 complex and silences Bax expression.

Here, our study showed that the SUZ12 protein is overexpressed in LUAD tissues and associated with worse clinicopathological features and a poor prognosis. SUZ12 promotes the malignant phenotype of LUAD cells, and it regulates many key signaling molecules. SUZ12 can directly bind to the Bax promoter. On the other hand, we should acknowledge that this study has certain limitations. We systematically investigated several aspects of the molecular mechanism of SUZ12. Since many target molecules are regulated by SUZ12, one aim of this study was to screen for the most suitable target molecule for promoter studies. Therefore, we did not investigate the expression of all molecules affected by SUZ12 overexpression, which were significant by SUZ12 knockdown. Nonetheless, our study may provide valuable insights into the molecular mechanisms of SUZ12 and potential development of innovative therapeutic target in LUAD.

## AUTHOR CONTRIBUTIONS

**Xingsheng Hu:** Data curation (lead); investigation (lead); methodology (lead); writing – original draft (lead). **Chunhong Hu:** Conceptualization (lead); supervision (lead). **Ping Zhong:** Data curation (equal); investigation (equal).

## FUNDING INFORMATION

Not applicable.

## CONFLICT OF INTEREST STATEMENT

The authors declare no conflicts of interest.

## ETHICS STATEMENT

The study of patients investigation (approval no. 2023‐015) and animal experiment (approval no. 20220820) were approved by the Ethics Committee of the Second Xiangya Hospital of Central South University, and patients' informed consent was waived.

## Supporting information


Figure S1.

Figure S2.

Figure S3.

Figure S4.

Figure S5.

Table S1.

Table S2.

## Data Availability

The data can be obtained from the corresponding author.
