# Supplementary material for: The expression and role of SUZ12 in lung adenocarcinoma
Source: Cancer Med. 2024 Oct 13;13(19):e70190. doi: 10.1002/cam4.70190 (PMC11471883; doi:10.1002/cam4.70190)
Supplement: Supplementary file 1 — Figure S1. [file CAM4-13-e70190-s006.pdf]

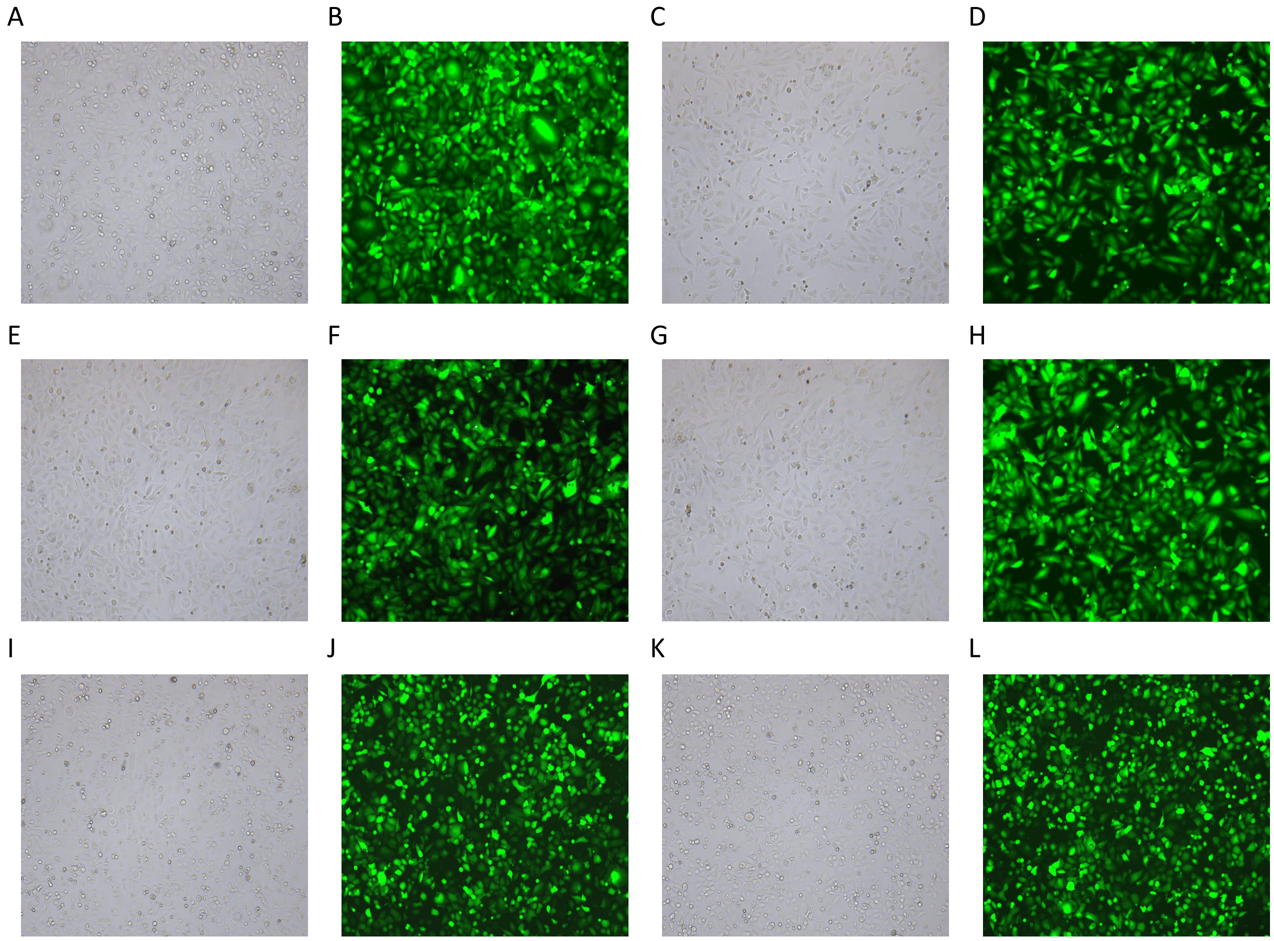

FIGURE S1.

The image of SUZ12 lentiviral vector transfection efficiency was detected by ordinary and fluorescent microscopy, left (white light) and right (fluorescence). sh-NC vector transfected A549 cells (A) and (B), sh-SUZ12-1 vector transfected A549 cells (C) and (D), sh-SUZ12-2 vector transfected A549 cells (E) and (F), sh-SUZ12-3 vector transfected A549 cells (G) and (H), oe-NC vector transfected NCI-H23 cells (I) and (J), and oe-SUZ12 vector transfected NCI-H23 cells (K) and (L). ×200.
