# Supplementary material for: The expression and role of SUZ12 in lung adenocarcinoma
Source: Cancer Med. 2024 Oct 13;13(19):e70190. doi: 10.1002/cam4.70190 (PMC11471883; doi:10.1002/cam4.70190)
Supplement: Supplementary file 2 — Figure S2. [file CAM4-13-e70190-s005.pdf]

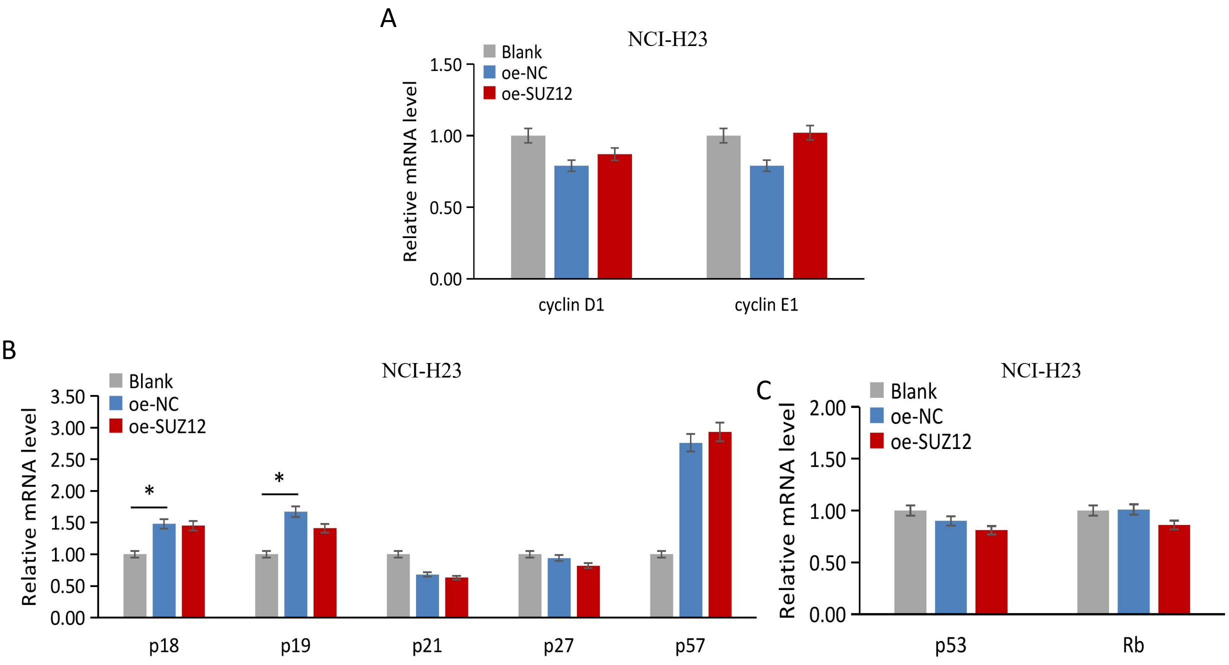

FIGURE S2.

The effect of oe-SUZ12 on the mRNA expression of cyclins, CKIs and p53/Rb was detected by qRT-PCR. oe-SUZ12 had no significant effect on the mRNA expression of cyclin D1/E1 (A), p18/19/21/27/57 (B) and p53/Rb (C).
