# Supplementary material for: The expression and role of SUZ12 in lung adenocarcinoma
Source: Cancer Med. 2024 Oct 13;13(19):e70190. doi: 10.1002/cam4.70190 (PMC11471883; doi:10.1002/cam4.70190)
Supplement: Supplementary file 3 — Figure S3. [file CAM4-13-e70190-s003.pdf]

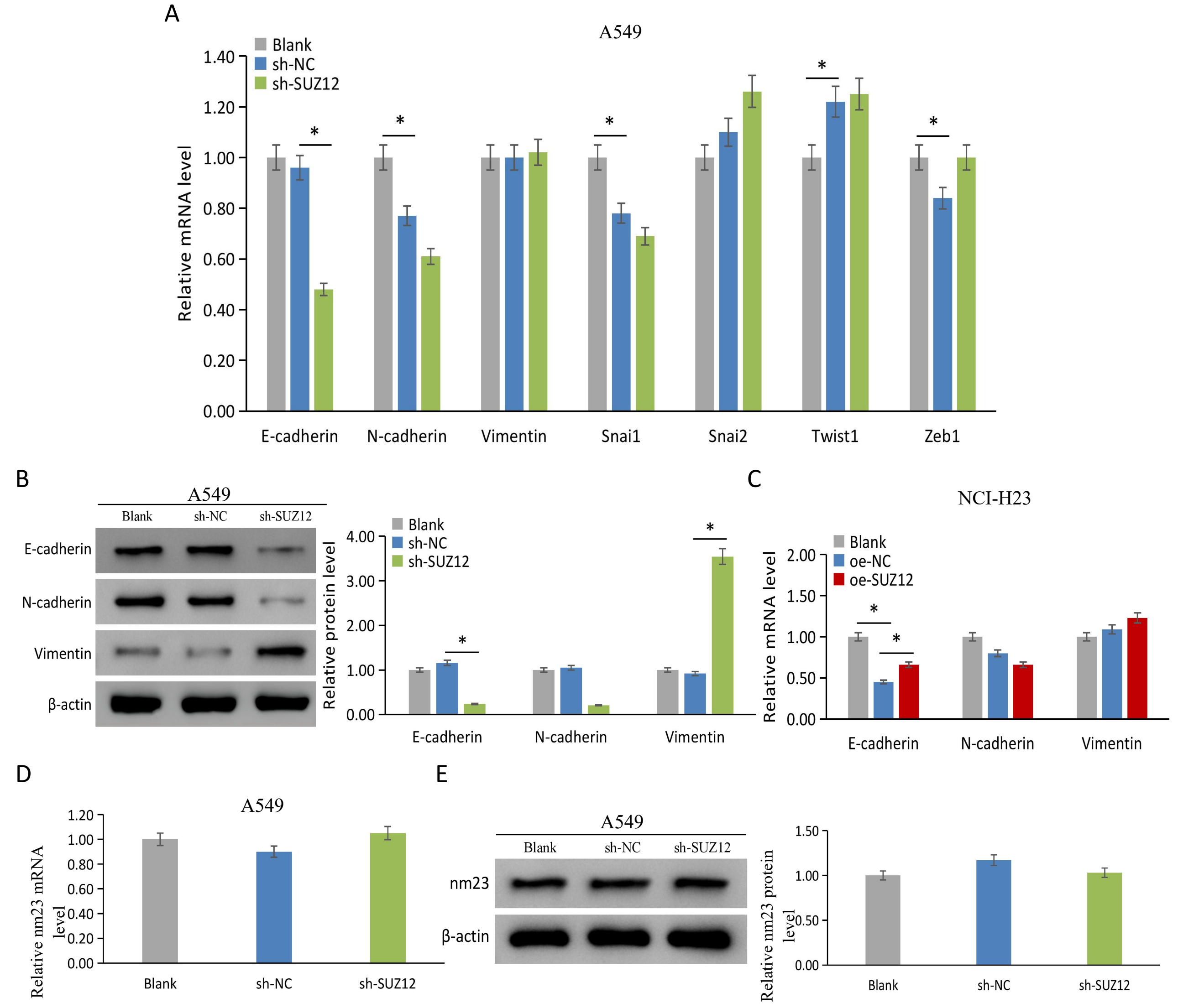

FIGURE S3.

The effect of SUZ12 on the expression of EMT markers and nm23 was detected by qRT-PCR and western blotting. sh-SUZ12 decreased E-cadherin mRNA (A) and protein (B) expression, while increased vimentin protein expression (B), without significantly effected nm23 mRNA (D) and protein (E) expression. oe-SUZ-12 increased E-cadherin mRNA expression (C).*P<0.05.
