# Supplementary material for: The expression and role of SUZ12 in lung adenocarcinoma
Source: Cancer Med. 2024 Oct 13;13(19):e70190. doi: 10.1002/cam4.70190 (PMC11471883; doi:10.1002/cam4.70190)
Supplement: Supplementary file 4 — Figure S4. [file CAM4-13-e70190-s004.pdf]

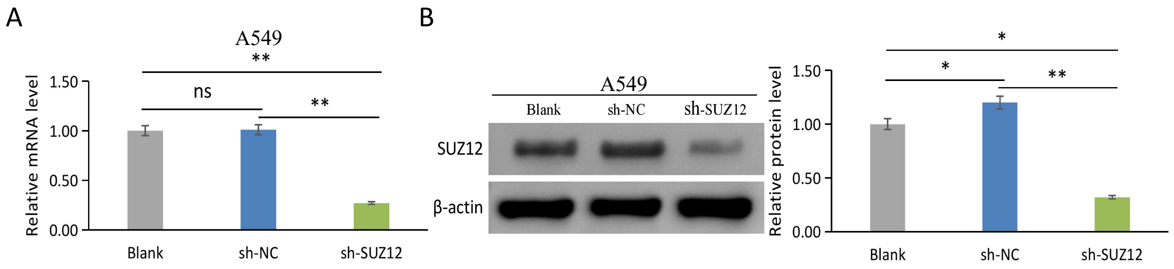

FIGURE S4.

The verification of sh-SUZ12/sh-NC stable transfection A549 cells was detected by qRT-PCR and western blotting. SUZ12 mRNA (A) and protein (B) in sh-SUZ12 group were significantly lower than that in sh-NC or blank groups. *P<0.05, **P<0.001, ns: no significance.
