# Supplementary material for: The expression and role of SUZ12 in lung adenocarcinoma
Source: Cancer Med. 2024 Oct 13;13(19):e70190. doi: 10.1002/cam4.70190 (PMC11471883; doi:10.1002/cam4.70190)
Supplement: Supplementary file 5 — Figure S5. [file CAM4-13-e70190-s007.pdf]

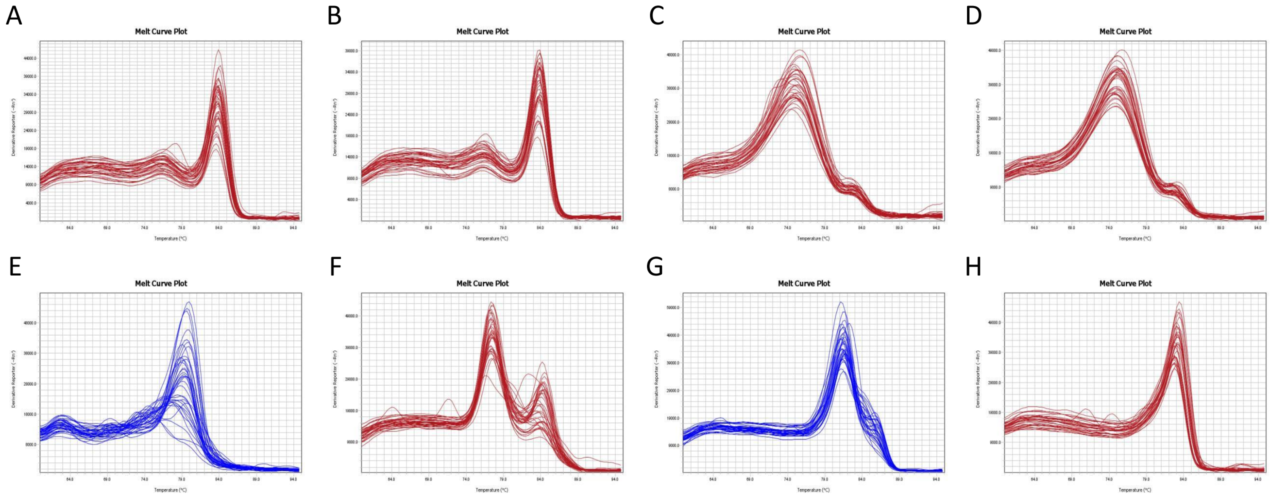

FIGURE S5.

The PCR product dissolution curves of ChIP primers was detected by qRT-PCR. The dissolution peaks of SUZ12 primer 1 (A), SUZ12 primer 2 (B), EZH2 primer 1 (E) and H3K27me3 primer (H) were all unimodal; the dissolution peaks of SUZ12 primer 3 (C), SUZ12 primer 4 (D), EZH2 primer 2 (F) and EZH2 primer 3 (G) were all bimodal.
