# Supplementary material for: The expression and role of SUZ12 in lung adenocarcinoma
Source: Cancer Med. 2024 Oct 13;13(19):e70190. doi: 10.1002/cam4.70190 (PMC11471883; doi:10.1002/cam4.70190)
Supplement: Supplementary file 6 — Table S1. [file CAM4-13-e70190-s002.docx]

Supplementary table 1. Primers of qRT-PCR

| Genes | Forward | Reverse |
| --- | --- | --- |
| GAPDH | ACAGCCTCAAGATCATCAGC | GGTCATGAGTCCTTCCACGAT |
| SUZ12 | ACATCAAAAGCTTGTCAGCTC | GCACCTGCTTTTTACCTGTGG |
| CDK2 | TTCTGCCATTCTCATCGGGTC | CTGGCTAGTCCAAAGTCTGCT |
| CDK3 | AGCGATTCCGCCATTGAGAA | TGCTTTGCTCTCCCCAGATG |
| CDK4 | GTGTATGGGGCCGTAGGAAC | CCATAGGCACCGACACCAAT |
| CDK6 | TGCCCACTGAAACCATAAA | ACCACAGCGTGACGACCA |
| cyclin D1 | ACCTCTTCACCTTATTCATGGCT | GCCTTTCCCGACCCTGCTAC |
| cyclin D2 | CAACCTGCTCCGAGACGAC | CTCACAGACCTCCAGCATCCAG |
| cyclin D3 | AACTTGGCTGAGCAGAGCAC | CATCCGAACAGAGCCAGTCT |
| cyclin E1 | TCTCCACCAAAGACAGTTGCG | CACTTTGCCCTGTTTGATGCC |
| P18 | ATCCCGATTTGAAAGACCGAAC | CCAGCAAAGTCTGTAAAGTGTCC |
| p19 | GAAGGTCCTAGTGGAGCACG | CACAGCAGTGTGACCCTCTT |
| P21 | TGTCCGTCAGAACCCATGCG | TCCCAACTCATCCCGGCCTC |
| P27 | TGGACCACGAAGAGTTAACCC | CCTCTTGCCACTCGTACTTGC |
| P57 | GCCAATTTAGAGCCCAAAGAGC | TGCTGCTACATGAACGGTCC |
| P53 | ACATTCTCCACTTCTTGTTCCCC | CTCCCCACAACAAAACACCAGT |
| Rb | AGAAGGTCTGCCAACACCA | ACGGTCGCTGTTACATACCAT |
| Bcl-2 | AGCTGCACCTGACGCCCTT | ACATCTCCCGGTTGACGCTCT |
| BAX | AGCTGCACCTGACGCCCTT | ACATCTCCCGGTTGACGCTCT |
| BAK | GACGACATCAACCGACGCTA | CCCCAATTGATGCCACTCTCA |
| Bid | GAAGTCCGGCCCCAGTACACA | GCCCTTAAATCACCTTCACACCT |
| BAD | GGTTCTGAGGGGAGACTGAGGT | TTCCTGCTCACTCGGCTCAA |
| CASP9 | AAGCCAACCCTAGAAAACCTTACCC | AGCACCGACATCACCAAATCCTC |
| MMP1 | GGGAGCAAACACATCTGACCT | TTCTCAATGGCATGGTCCACA |
| MMP2 | GCACCACTGAGGACTACGAC | TGCAGCTCTCATATTTGTTGCC |
| MMP3 | GAGGACACCAGCATGAACCT | CTTCCCCGTCACCTCCAATC |
| MMP9 | CTGAAGGCCATGCGAACCCCA | GCAAAGGCGTCGTCAATCACC |
| MMP14 | CCCAACATCTGTGACGGGAA | TTGGTTATTCCTCACCCGCC |
| TIMP1 | GTGGCACTCATTGCTTGTGG | CAAGGTGACGGGACTGGAAG |
| TIMP2 | CATCGTGGAAGCATTTGACCC | GCCACACCTTTCAGACCGAAC |
| TIMP3 | GACACTACCCTTCCATGCCC | GTGCCATCTTGGGGTAGTCC |
| TIMP4 | GCCTCCCAAACCCCATTAGT | TCGCCATTTCTCCCCTACCA |
| E-cadherin | TCAGTACAACGACCCAACCC | GCCTTCCTACAGACGCCAG |
| N-cadherin | TGCCCCTCAAGTGTTACCTC | CAAAATCACCATTAAGCCGAGT |
| Vimentin | CCCTTGACATTGAGATTGCCACC | ACCGTCTTAATCAGAAGTGTCCT |
| Snai1 | AAGATGCACATCCGAAGCCA | CATTCGGGAGAAGGTCCGAG |
| Snai2 | AGGACACATTAGAACTCACACGG | TACACAGCAGCCAGATTCCTC |
| Twist1 | CCAGGTCCTCCAGAGCGACGAG | CCATCCTCCAGACCGAGAAGGC |
| ZEB1 | GGAATCAACTGTTCAACCCCTT | ACATTACCATCTACCGCCACT |
| ITGAV | GGTCTTTCTACCTCTGCCTCAC | CGAGAAGAAACATCCGGCAC |
| ITGA5 | TTCGGTTTACAGTCCCTCATC | GCTTTGCGAGTTGTTGAGATT |
| ITGB1 | CAAGAGAGCTGAAGACTATCCCA | TGAAGTCCGAAGTAATCCTCCT |

Supplementary table 1. Primers of qRT-PCR (continued)

| Genes | Forward | Reverse |
| --- | --- | --- |
| ITGB3 | CTTTTGCCATCCCCTCAACCC | GCCCAGACATCCTCACTCCC |
| ITGB5 | CTCGGAGCCCAAGTCGC | GGACTCCTAGTGTGTCGCC |
| nm23 | TACCATCCCCGACCATCTGA | TCTATTAGGTCAGGTTATTCAGTGT |
| PD-L1 | ACAATTAGACCTGGCTGCACT | ACCCCTGCATCCTGCAATTT |
