# Supplementary material for: The expression and role of SUZ12 in lung adenocarcinoma
Source: Cancer Med. 2024 Oct 13;13(19):e70190. doi: 10.1002/cam4.70190 (PMC11471883; doi:10.1002/cam4.70190)
Supplement: Supplementary file 7 — Table S2. [file CAM4-13-e70190-s001.docx]

Supplementary table 2. Primers of ChIP

| Primer | Forward | Reverse |
| --- | --- | --- |
| SUZ12-primer1 | TTGGAGTTTCCCTCTTGTT | TGAAACCCTATCCCTACTAAA |
| SUZ12-primer2 | GACCAGCCTGACCAACATA | CTCTTGTTGCCCAGGATG |
| SUZ12-primer3 | ATCCTGGGCAACAAGAGC | TAAAAAGTGGCAGAAACTAATC |
| SUZ12-primer4 | CTGCTGGCACTTATCGG | GCTGAACGTGCGTCCTT |
| EZH2-primer1 | TTTCTGGGAGAATTGAGG | TCGTTTGAATCTGGAGGC |
| EZH2-primer2 | GCCCAAACCCTTTATCAT | TGGGATTACAGGCGTGAG |
| EZH2-primer3 | GCAGTGAGCCATGATTGG | CCGTCTCAGCCTTCCAAA |
| H3K27me3-  primer1 | TGCCCGAAACTTCTAAAA | CGTGACTGTCCAATGAGC |
